# Identification and characterization of chromosomal *relBE* toxin-antitoxin locus in *Streptomyces cattleya* DSM46488

**DOI:** 10.1038/srep32047

**Published:** 2016-08-18

**Authors:** Peng Li, Cui Tai, Zixin Deng, Jianhua Gan, Marco R. Oggioni, Hong-Yu Ou

**Affiliations:** 1 State Key Laboratory of Microbial Metabolism, Joint International Laboratory on Metabolic & Developmental Sciences, School of Life Sciences & Biotechnology, Shanghai Jiao Tong University, Shanghai, China; 2 Department of Physiology and Biophysics, School of Life Sciences, Fudan University, Shanghai, China; 3 Department of Genetics, University of Leicester, Leicester, UK

## Abstract

The *relBE* family of Type II toxin-antitoxin (TA) systems have been widely reported in bacteria but none in *Streptomyces*. With the conserved domain searches for TA pairs in the sequenced *Streptomyces* genomes, we identified two putative *relBE* loci, *relBE1sca* and *relBE2sca*, on the chromosome of *Streptomyces cattleya* DSM 46488. Overexpression of the *S. cattleya* toxin RelE2sca caused severe growth inhibition of *E. coli* and *S. lividans,* but RelE1sca had no toxic effect. The toxicity of RelE2sca could be abolished by the co-expression of its cognate RelB2sca antitoxin. Moreover, the RelBE2sca complex, or the antitoxin RelB2sca alone, specifically interacted with the *relBE2sca* operon and repressed its transcription. The *relBE2sca* operon transcription was induced under osmotic stress, along with the ClpP proteinase genes. The subsequent *in vivo* analysis showed that the antitoxin was degraded by ClpP. Interestingly, the *E. coli* antitoxin RelBeco was able to alleviate the toxicity of *S. cattleya* RelE2sca while the mutant RelB2sca(N61V&M68L) but not the wild type could alleviate the toxicity of *E. coli* RelEeco as well. The experimental demonstration of the *relBEsca* locus might be helpful to investigate the key roles of type II TA systems in *Streptomyces* physiology and environmental stress responses.

Bacterial toxin-antitoxin (TA) system is originally identified in low copy number plasmids and shown to maintain the plasmid stability by post-segregational killing of plasmid-free daughter cells^1^. In recent years, bioinformatics and experimental evidence show that the type II TA modules are widely spread not only upon plasmids but also on chromosomes^2^,^3^. The TA loci typically consist of two but occasionally three tandem genes. The toxin genes invariably code for proteins, while matching antitoxin genes code for either antisense RNA or antitoxin proteins, resulting in classification as type I or type II TA loci, respectively. The chromosomal type II TA loci have been either demonstrated or hypothesized to play key roles in the stabilization of horizontally acquired genetic elements^4^, stress responses^5^, and traits in bacterial physiology such as the programmed cell death^6^ and persister cell formation^7^.

A number of functionally distinct type II TA systems have been identified by using experimental and bioinformatics approaches. As of June 2015, TADB, the web-based toxin-antitoxin database managed by our group, has collected 6,156 putative TA loci in 679 bacterial and archaeal genomes^8^; interestingly, 214 of the collected TA loci had been assigned to the *relBE* family. *relBE* is one of the best-documented type II TA loci with detailed reports about transcription regulation, toxin activity, antitoxin degradation and the TA complex formation^9^. Many of the toxins encoded on the chromosomes have been found to interfere with the protein synthesis in either a ribosome-dependent^10^,^11^ or ribosome-independent manner^12^,^13^. Under non-stress conditions, the toxicity of RelE is neutralized by the antitoxin RelB by forming a tight non-toxic RelBE complex^14^. The concentrations of toxin and antitoxin in the cells are regulated by the antitoxin or TA complex that is capable of repressing the transcription of the TA operon by binding specifically to the promoter. In some cases, the repression is further strengthened by conditional cooperativity^15^. Under environmental stress conditions, the antitoxin is degraded by cellular proteinases, such as the ATP-dependent Lon proteinases in *E. coli*^16^.

As listed in TADB, 169 out of 6,156 TA loci have been experimentally characterized, including for example 13 out of 16 type II TA pairs in *E. coli* K-12 MG1655, 5 out of 18 in *Salmonella enterica* Typhimurium LT2 and 33 out of 77 in *Mycobacterium tuberculosis* H37Rv^8^. However, there are few reports on the TA systems encoded by *Streptomyces*, the largest genus of *Actinobacteria*. To date, only one functional TA system, the *yefM-yoeB* locus on the *S. lividans* TK24 chromosome^17^, has been experimentally demonstrated in *Streptomyces. Streptomyces* are famous for producing many bioactive secondary metabolites, such as antibiotics^18^. Their complex life cycle that contains a vegetative and a spore stage made them excellent model organisms for studying prokaryotic differentiation^19^,^20^. The responses to nutrient limitation or other physiological stresses, including ppGpp, had been shown to play important roles in the differentiation process of *Streptomyces*^21^. It is worth noticing that ppGpp is also able to control bacterial persistence by induction of TA activity^22^. Availability of the complete genome sequences of *Streptomyces* allowed us to *in silico* identify the putative type II TA systems present in this genus. TADB had archived 22 putative TA loci in *S. coelicolor* A3(2), 27 in *S. avermitilis* MA-4680 and 14 in *S. griseus* NBRC 13350; however, none *relBE* family TA locus was found. We thus searched for the *relBE* locus in the completely sequenced *Streptomyces* genomes based on the conserved RelBE domains. Two putative *relBE* loci were obtained on the linear chromosome of *Streptomyces cattleya* DSM 46488.

The *S. cattleya* DSM 46488 strain is unusual in its ability to synthesize fluorine-containing natural products, including fluoroacetate and 4-fluorothreonine. We had completely sequenced its genome and two linear replicons were found^23^, a 6.3-Mb chromosome (GenBank accession no. CP003219) and a 1.8-Mb mega-plasmid (CP003229). In this study, we experimentally investigate the two putative *relBE* loci identified on the linear chromosome of *S. cattleya* DSM46488. The toxin homologous protein RelE2sca (SCATT_39270) and the antitoxin homologous protein RelB2sca (SCATT_39280) were found to be organized as an operon. The over-expression of RelE2sca resulted in the cell growth inhibition of both *E. coli ΔrelBE* mutant and *S. lividans*, and the co-expression of the cognate RelB2sca could counteract the toxicity. RelB2sca may be degraded by the ClpP proteinases under osmotic stress. Interestingly, the antitoxin RelBeco (b1564) from *E. c oli* K-12 MG1655 can also alleviate the toxicity of *S. cattleya* RelE2sca.

## Results

### Two putative *relBE* TA loci were predicted in the *S. cattleya* DSM46488 chromosome

**Figure 1:**
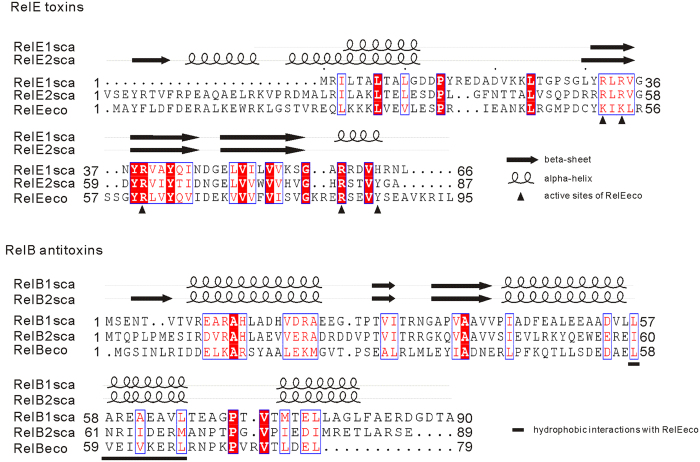
Amino acid sequence alignments for three RelE toxin proteins and their cognate RelB antitoxin proteins. Putative RelBE1sca (SCATT_20920-SCATT_20930) and RelBE2sca (SCATT_39280-SCATT_39270) pairs were identified on the *S. cattleya* DSM46488 chromosome while the well-documented RelBE module RelBEeco (b1564-b1563) is encoded by *E. coli* K-12 MG1655 chromosome. Conserved residues are shown in red and by boxed. The black triangle indicates the active sites of the *E. coli* toxin RelEeco while the underlined sequence indicates the RelBeco conserved motif interacting with RelEeco^14^,^30^. Secondary structures were predicted by using PSIPRED^43^.

The putative type II TA pairs were detected based on typical TA features, including those of the conserved TA domains and a pair of two short genes organized as an operon structure. Thirty-three putative type II TA loci were annotated in the completely sequenced *S. cattleya* DSM46488 genomes (supplementary Table S1), including two *relBE* homologs on its linear chromosome, *SCATT_20920-SCATT_20930* and *SCATT_39280-SCATT_39270*, named *relBE1sca* and *relBE2sca*, respectively. The *S. cattleya* toxin proteins RelE1sca (SCATT_20930) and RelE2sca (SCATT_39270) exhibits low sequence similarities to the well documented RelE toxin b1563 (RelEeco) of *E. coli* K-12 MG1655 with 23% and 28% BLASTp identities, respectively; however, four conserved motifs were found among these RelE homologs, which were predicted to form two alpha-helixes and two beta-sheets (Fig. 1). Similarly, four alpha-helixes and two beta-sheets were also present in the three cognate RelB antitoxins, RelB1sca (SCATT_20920) and RelB2sca (SCATT_39280) of *S. cattleya*, and RelBeco (b1564) of *E. coli*.

### *relE*2*sca* and its upstream gene *relB2sca* were organized into an operon

**Figure 2:**
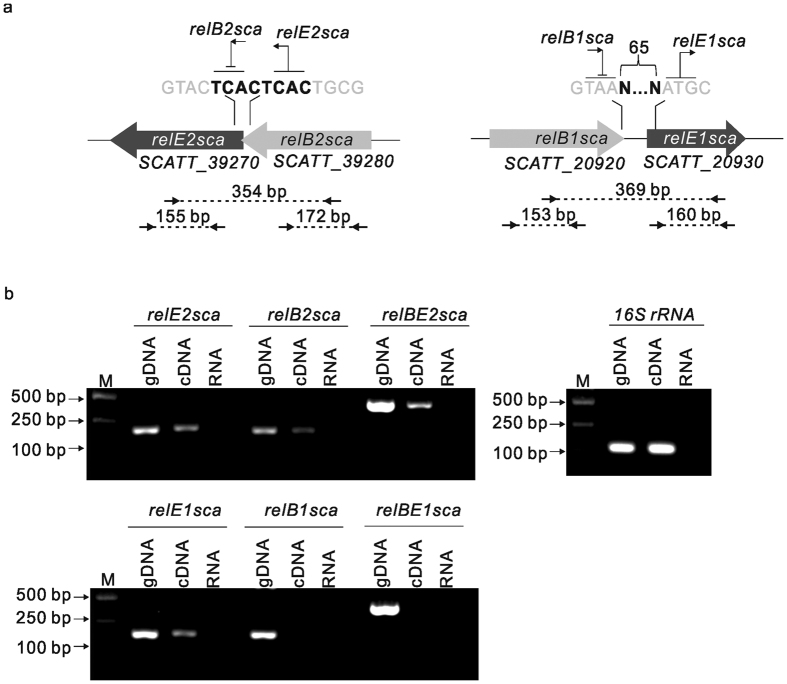
Genetic organization of the two putative *relBE* loci on *S. cattleya* DSM46488 chromosome: *relBE1sca* (*SCATT_20920-SCATT_20930*) and *relBE2sca* (*SCATT_39280-SCATT_39270*). (**a**) Scaled schematic representation of the toxin and antitoxin genes. The overlapping or separate region is shown in bold. The arrows indicate the primers used in the transcription analysis and the numbers indicate the expected size of the PCR products; (**b**) Transcription analysis of PCR amplification of *S. cattleya relBE* genes using cDNA and gene-specific primers to amplify from the 3′ end of *relBs* to the 5′ end of *relEs* (spanning both the protein-coding and the intergenic region). Lane M indicates the standard DNA size marker. Each set of the three lanes consisted of positive controls using genomic DNA as template (gDNA). PCR amplified products using cDNA prepared from log-phase *S. cattleya* (cDNA) and negative controls using the total RNA without reverse transcriptase (RNA). 16S rRNA was used as the positive control. The gels were cropped from the original images available at the supplementary Fig. S10.

Toxin-antitoxin modules are typically organized into operons with the antitoxin genes located upstream of the toxin genes, often overlapping or with a small intergenic region between the two genes. As seen in Fig. 2a, the putative toxin gene *relE2sca* is overlapped by its upstream antitoxin gene *relB2sca* by 8 nucleotides, while relE1sca and relB1sca were separated by a 65-bp region. To determine whether the two putative *relBE* gene pairs were cotranscribed, respectively, and thus formed individual operons, total RNA of logarithmic phase *S. cattleya* DSM46488 was isolated and converted to cDNA by reverse transcriptase. The cDNA was PCR amplified by using specific primers spanning the toxin and antitoxin genes (indicated by small arrows in Fig. 2a). A band with the expected size was obtained for *relBE2sca*, but not for *relBE1sca*. mRNAs of the individual toxin genes and antitoxin genes were detected as well, and we also succeed in obtaining the expected bands except for *relB1sca*. Genomic DNA contamination was eliminated by using RNA without reverse transcriptase as the template. Likewise, as a positive control, bands with expected sizes were observed for 16S rRNA amplification by using cDNA and for all amplifications by using *S. cattleya* DSM46488 genomic DNA as the template (Fig. 2b). These data suggested that *relBE2sca* was organized into an operon.

### Overexpression of *S. cattleya* toxin RelE2sca was lethal in *E. coli* and *S. lividans*

**Figure 3:**
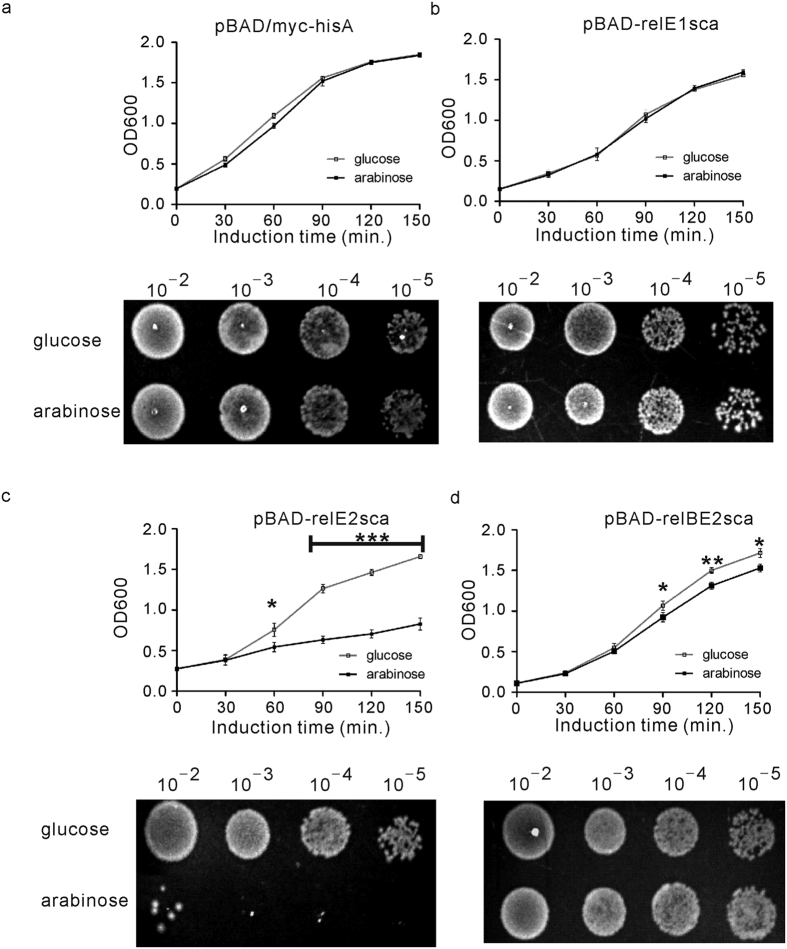
Effect of over-expression of the putative toxin proteins RelE1sca and RelE2sca on the growth of *E. coli* MGJ5987 (MG1655 ∆*relBE* mutant). *E. coli* MGJ5987 cells transformed with individual plasmids were grown in LB medium until the OD_600_ reached 0.2: (**a**) control plasmid (pBAD/myc-hisA), (**b**) plasmid carrying *relE1sca* (pBAD-relE1sca), (**c**) plasmid carrying *relE2sca* (pBAD-relE2sca) and (**d**) plasmid carrying the intact *relBE2sca* operon (pBAD-relBE2sca). Then, at time zero, 0.2% glucose (the hollow square) was added into one-half of each culture and 0.2% L-arabinose (the solid square) was added into the other half. Cell growth was monitored by measuring the OD600 every 30 minutes. The means and standard deviation of three different experiments are shown. For statistical analysis, two-way analysis of variance with Bonferroni post-tests were used to obtain P values for each time point: *P < 0.05; **P < 0.01; ***P < 0.001. 3 μl of serial dilutions of different cultures which were collected after induction by glucose or arabinose for 1 hour were spread onto the LA plate and incubated at 37 °C for 12 hours.

To determine whether the putative toxins RelE1sca and RelE2sca of *S. cattleya* were functional or not, the effect of the overexpression of their corresponding genes was assessed in *E. coli* MGJ5987 (MG1655 *ΔmazF ΔchpB ΔrelBE Δ(dinJ-yafQ) Δ(yefM-yoeB) ΔhigBAΔ(prlF-yhaV) ΔyafNO ΔmqsRA ΔhicAB*)^24^. The toxin genes *relE1sca* and *relE2sca* were firstly cloned into the *E. coli* expression vector pBAD/myc-hisA and placed under the control of arabinose inducible promoter P_BAD_, producing plasmids pBAD-relE1sca and pBAD-relE2sca, respectively (supplementary Table S2). Then, the growth of *E. coli* MGJ5987 strains containing the corresponding plasmid was used to assess the effects of RelE1sca or RelE2sca. Consequently, the growth of *E. coli* cells transformed with pBAD-relE2sca was strongly inhibited in the presence of 0.2% L-arabinose that induced the overexpression of *relE2sca* (Fig. 3c), while the normal growth was observed in the presence of 0.2% glucose. The difference in the number of *E. coli* colonies obtained between one-hour induction of L-arabinose and glucose was also evident when grown on Luria agar (LA) plates (Fig. 3c). This suggested that overexpression of *S. cattleya* toxin RelE2sca is lethal in *E. coli*. However, there was no significant difference in growth observed for the cells harboring pBAD-relE1sca or the blank plasmid pBAD-myc/hisA either in the presence of L-arabinose or glucose (Fig. 3a,b), indicating that RelE1sca had no toxic effect to *E. coli ΔrelBE* mutant under the condition studied.

The toxicity of RelE2sca was predicted to be neutralized by the upstream cognate antitoxin protein RelB2sca. To balance the protein expression levels of the toxin and antitoxin, the *relBE2sca* region of *S. cattleya* was cloned into pBAD/myc-hisA, resulting in pBAD-relBE2sca (supplementary Table S2). As predicted, normal growth of *E. coli* MGJ5987 harboring plasmid pBAD-relBE2sca in the Luria-Bertani (LB) broth supplemented with arabinose or glucose was observed due to co-expression of RelB2sca and RelE2sca (Fig. 3d). Similarly, after induction by arabinose or glucose for one hour, the cells with different dilutions were spread onto the agar plates and showed the similar results (Fig. 3d). In addition, the direct interaction between the toxin RelE and antitoxin RelB to form the RelBE complex was examined. RelB2sca and RelE2sca-His_6_ were coexpressed in *E. coli* BL21(DE3) and co-purified. As shown in supplementary Fig. S1a, the 11.54-kDa and 10.32–kDa proteins, consistent with the expected molecular masses of the recombinant RelB2sca and RelE2sca-His_6_, respectively, were co-purified under native conditions. The 11.54–kDa protein (RelE2sca-His_6_) was purified under denaturing conditions whereas the 10.32–kDa protein (RelB2sca) was obtained from the complex under denaturing condition. The complex was finally subjected to the size-exclusion chromatography, showing that the ratio of RelB2sca and RelE2sca-His_6_ was 2:2 (supplementary Fig. S1b). These results all suggested that the *S. cattleya* toxin RelE2sca was lethal to *E. coli* and its toxicity could be abolished by RelB2sca. All of the results above suggested that *relBE2sca* might represent a functional toxin-antitoxin module while *relBE1sca* has no activity under the condition studied.

Results of heterologous expression experiments of RelBE2sca in *Streptomyces* were consistent with those in *E. coli* mentioned above (supplementary Fig. S2). *S. lividans* TK24, which was predicted to contain no homolog of RelB or RelE, was used as the host to assess the RelE2sca toxicity. The *S. cattleya* RelBE protein-coding regions, *relE2sca*, *relB2sca* and *relBE2sca*, were cloned into the *Streptomyces - E. coli* shuttle vector pIB139 (integrative plasmid) under the control of the constitutive promoter PermE (supplementary Table S2). Subsequently, the resulting pIB139-relE2sca was unable to be introduced into *S. lividans* TK24 while both pIB139-relB2sca and pIB139-relBE2sca transferred at high frequency (supplementary Fig. S2). It suggested that the expression of the toxin RelE2sca was lethal in *Streptomyces* but the antitoxin RelB2sca could counteract this toxic effect. Additionally, the expression of another toxin RelE1sca had no toxic effect on the growth of *S. lividans* TK24 under the condition studied (supplementary Fig. S2).

### The *relBE2sca* operon was auto-regulated by the RelBE2sca TA complex

**Figure 4:**
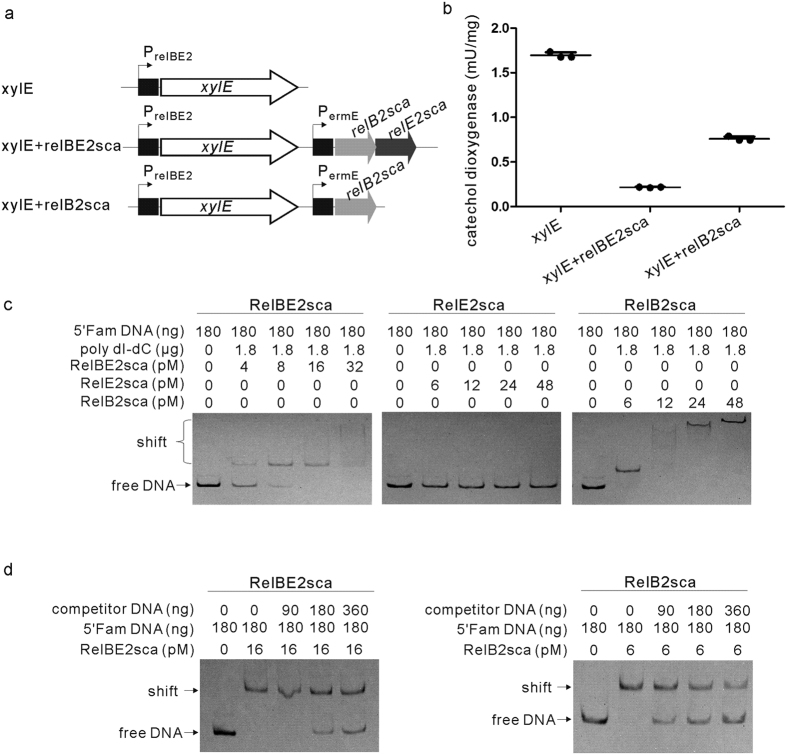
Auto-regulation of the *S. cattleya relBE2sca* operon by the RelBE2sca TA complex or the antitoxin RelB alone. (**a**) Schematic representation of *in vivo* regulation of *relBE2sca* operon in *S. lividans* TK24 *xylE,* reporter gene coding for catechol 2,3-dioxygenase; P_*relBE2*_, promoter region of *S. cattleya relBE2sca*; P_*ermE*_, strong constitutive promoter. (**b**) Activity of catechol 2,3-dioxygenase was assayed by monitoring the increase in absorbance at 375 nm (A375) every 10 minutes for the *S. lividans* TK24 cells carrying the resulting plasmids. (**c**) Analysis of the interactions between P_*relBE2*_ and RelBE2sca proteins by using EMSA *in vitro*. Increasing the amount of RelBE2sca-His_6_, RelE2sca-His_6_ or RelB2sca was incubated with the promoter labeled with 5′-FAM. Poly dI-dC was used to inhibit the unspecific interaction. Unbound DNA fragments were separated from protein-DNA complex by electrophoresis in 6% native PAGE gel. (**d**) Increasing amounts of unlabeled competitor DNA were added into the reaction system in which the amounts of His6-RelBE2sca complex or RelB2sca were set at 16 pM or 6 pM, respectively.

It has been reported that the transcription of the *E. coli relBE* operon was specifically repressed by the overexpression of its encoded TA complex or antitoxin alone. Here the reporter gene *xylE* (encoding catechol 2,3-dioxygenase) was used to investigate whether the transcription of *S. cattleya relBE2sca* operon was regulated *in vivo* by the RelBE2sca complex or by RelB2sca in *Streptomyces*^25^. First, the intergenic region containing the promoter region of *relBE2sca* of *S. cattleya* (P_*relBE2*_) was amplified and placed upstream of the promoterless *xylE* gene to control the expression of *xylE* (Fig. 4a). Then, the protein-coding fragments of *relBE2sca* and *relB2sca* were amplified and placed under the control of the strong constitutive promoter PermE, respectively. Finally, cultures of *S. lividans* TK24 carrying the resulting plasmids were assayed for catechol dioxygenase activity. Figure 4b showed the results of three independent determinations. In the cases of the high gene expressions of the RelBE2sca complex and the antitoxin RelB2sca, catechol dioxygenase activities were consistently lower than activities obtained with XylE alone, by about 8-fold and 2-fold, respectively. It suggested that the transcription of *S. cattleya relBE2sca* module was inhibited by the overexpression of the antitoxin RelB2sca alone, and more efficiently by the TA complex RelBE2sca, which was in agreement with data in *E. coli*.

Furthermore, the specific interaction between the P_*relBE2*_ region and the purified RelBE2sca-His_6_ complex was assessed by Electrophoretic Mobility Shift Assay (EMSA) *in vitro*. The 5′-FAM-labeled P_*relBE2*_region was amplified from *S. cattleya* DSM46488 genomic DNA by using 5′-FAM oligonucleotides (supplementary Table S2). The retarded bands were observed when the FAM-labeled DNA probe were incubated with increasing amounts of purified RelBE2sca-His_6_ complex or RelB2sca alone, but not with RelE2sca alone (Fig. 4c). The signal of the retarded bands decreased with the increased unlabeled specific competitor DNA probes (Fig. 4d). In addition, the binding sites of RelBE2sca-His_6_ complex to the labeled probes were located precisely by DNase I footprinting (supplementary Fig. S3a). The DNA probes were the same as those used in the EMSA experiments. The DNA sequencing electropherogram showed that the 14-bp DNA fragment (GTACACCTGAGACA) upstream of the putative -35 region of P_*relBE2*_was clearly protected by the RelBE2sca-His_6_ complex (supplementary Fig. S3a). This was also supported by the fact that the retarded band disappeared when the RelBE2-His_6_ complex was incubated with the mutant probe, in which the protected DNA sequence was deleted (supplementary Fig. S3b). Together, these observations indicated that the RelBE2sca complex repressed the transcription of the *relBE2sca* operon by interacting specifically with the 14-bp DNA fragment within the P_*relBE2*_ region.

### Transcription of the antitoxin *relB2sca* was enhanced under osmotic stress condition

**Figure 5:**
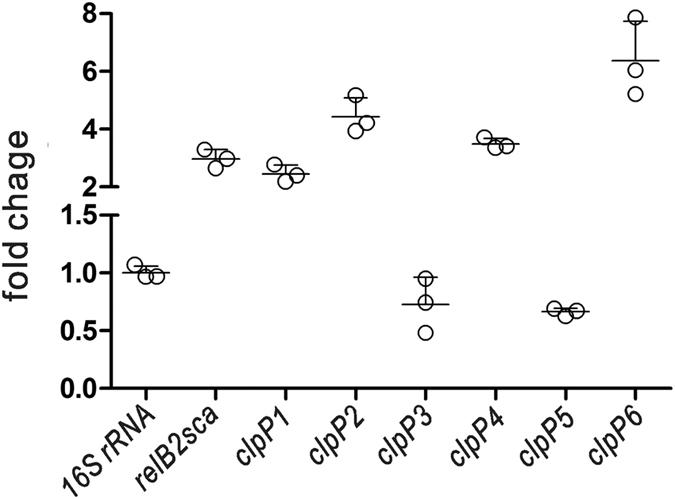
Transcription of the antitoxin gene *relB2sca* and the six *clpP* proteinases in *S. cattleya* under osmotic stress. After culturing in minimal medium to log phase, *S. cattleya* DSM46488 cells were exposed to 0.5 M NaCl and 0.5 M sucrose for 3 hours. Cells were harvested and RNA was isolated and amplified. Gene transcription levels were measured by using real-time quantitative PCR and the 16S *rRNA* was used as a control. The experiments were repeated three times, and the error bars represented the standard deviation.

To determine whether the *relBE*2sca operon was able to respond to the environmental stress like the typical RelBE system reported in *E. coli*, the transcriptional level of the antitoxin gene *relB2sca* was measured under osmotic stress as described in the Methods section. The *relB2sca* mRNA level was evaluated by a real-time quantitative PCR (qRT-PCR) assay, showing that the RelB2sca mRNA levels were increased 3 folds (Fig. 5). The result above indicated that the osmotic stress could induce the transcription of *relBE2sca*. In addition, the *relB2sca* transcription level also increased under the other environmental stresses tested, including high temperature, strong amino acid starvation, glucose starvation and antibiotic treatment (supplementary Fig. S4).

### Antitoxin protein RelB2sca was degraded by ClpP proteinases

**Figure 6:**
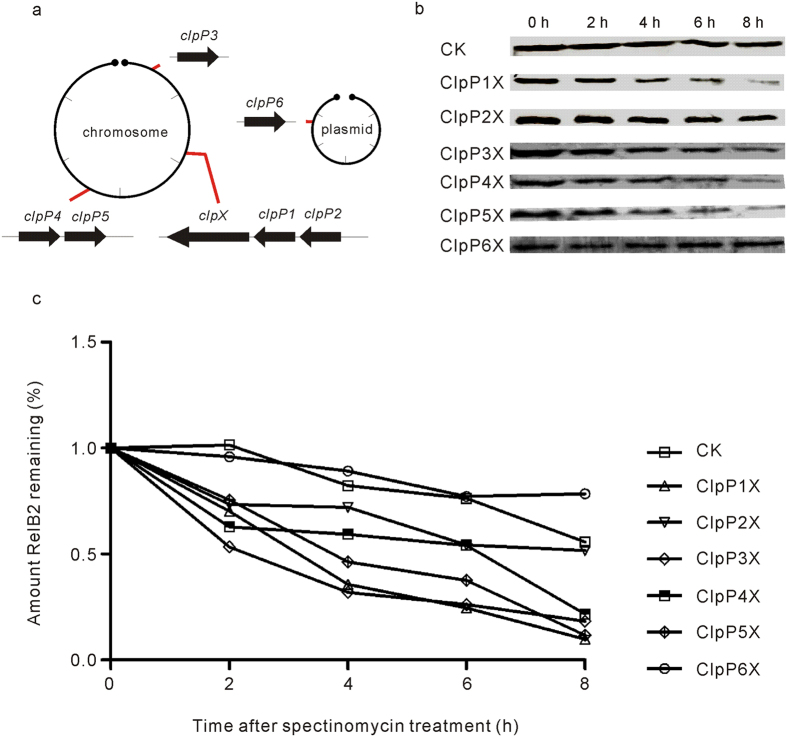
Intracellular stability of the *S. cattleya* antitoxin protein RelB2sca in the absence or presence of the proteinase ClpPnX (n = 1, 2, 3, 4, 5 or 6) detected by Western blotting. The strains *E. coli* BL21(DE3)/pLysS with corresponding plasmids were grown in LB broth in the presence of 0.2% L-arabinose to induce the RelB2sca expression. Then 0.5 mM IPTG was added to induce the proteinases ClpPnX expression. One hour later, 200 μg/ml spectinomycin was added to inhibit protein synthesis. Samples were collected every two hours. The levels of the antitoxin protein RelB2sca were monitored by Western blotting analysis. (**a**) Six ClpP proteinase gene loci and one chaperone ClpX gene locus found on the linear chromosome and mega-plasmid of *S. cattleya* DSM46488. (**b**) RelB2sca protein band of Western blotting (CK denoted the control). (**c**) graph described the percentage of a signal for RelB2sca.

The labile antitoxin RelB in *E. coli* has been reported to be sensitive to the ATP-dependent proteinase Lon^16^, allowing the stable toxin RelE to be released and to carry out the toxic effect. Interestingly, in *Synechocystis* sp. PCC 6803, RelB was also found to be degraded by the ClpP proteinase with the ClpX or ClpA as the chaperon^26^. We thus investigated whether the *S. cattleya* ClpP proteinase was involved in the proteolysis of the antitoxin RelB2sca. Six putative ClpP proteinase homologs have been annotated in the *S. cattleya* DSM46488 genome (Fig. 6a), including five found on the linear chromosome (ClpP1, SCATT_17340; ClpP2, SCATT_17350; ClpP3, SCATT_03550; ClpP4, SCATT_32700; ClpP5, SCATT_32710) and one in the mega-plasmid (ClpP6, SCATT_p13740). One chaperone gene *clpX* (*SCATT_17330*) was also found to be clustered with the proteinase genes *clpP1-clpP2*. Under the osmotic stress, the transcription levels of 4 out of 6 ClpP proteinase genes increased significantly, along with the antitoxin gene *relB2sca* (Fig. 5 and supplemental Fig. S4). The transcription levels of *clpP1*, *clpP2*, *clpP4* and *clpP6* increased by 2 folds, 4 folds, 3 folds and 6 folds, respectively. As for *relBE2sca*, the transcription levels of ClpP proteinase genes were also determined whether they were induced by the other environmental stresses tested (supplementary Fig. S4). Diverse ClpP proteinase genes exhibited different transcription levels under these stresses. These data are in accordance with the hypothesis that the ClpPs might hydrolyze RelB2sca and participate in the activation of the *relBE2sca* module.

To determine whether the antitoxin RelB2sca was degraded by the ClpP proteinases of *S. cattleya* DSM46488, the *relB2sca* gene was firstly amplified and cloned into pBAD/myc-hisA. resulting in pBADAHis which expressed the antitoxin with C-terminal *His*6-*tag* (supplementary Table S2). The individual proteinase genes *clpPs* and the chaperone gene *clpX* were also cloned into the MCS1 and MCS2 of *E. coli* expression vector pRSFDuet under the control of the T7 promoter, respectively. Then, the *intracellular stability* of the antitoxin *protein* RelB2sca, which was coexpressed with ClpP1X, ClpP2X, ClpP3X, ClpP4X, ClpP5X and ClpP6X, was investigated by Western blotting analysis. The results showed that the half-life of RelB2sca coexpressed with ClpP1X, ClpP3X, ClpP4X and ClpP5X was significantly shorter than that of RelB2sca expressed alone in *E. coli* (Fig. 6b,c), indicating that the proteinases ClpPs of *S. cattleya* DSM46488 were indeed able to degrade the antitoxin RelB2sca *in vivo*.

### Toxic effect of *S. cattleya* RelE2sca was alleviated by co-expression of *E. coli* antitoxin RelBeco

**Figure 7:**
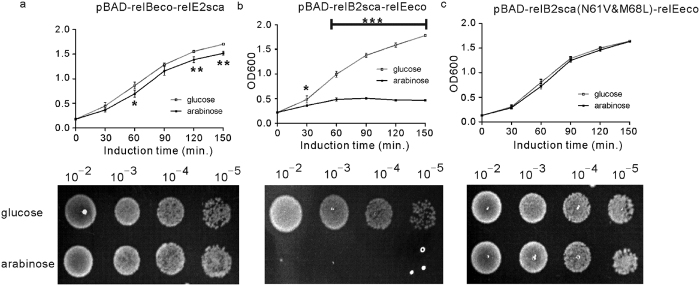
Homologous RelBE toxin-antitoxin protein interactions between *S. cattleya* and *E. coli*. Overnight cultures of *E. coli* MGJ5987 (MG1655 ∆*relBE* mutant) carrying individual plasmids were diluted 1:100 in LB broth supplemented with 50 μg/ml ampicillin: (**a**) pBAD-relBsco-relE2sca, (**b**) pBAD-relB2sca-relEeco, and (**c**) pBAD-relB2sca(N61V&M68L)-relEeco. When the OD_600_ reached to about 0.2, each culture was divided into two equals. 0.2% glucose (hollow square) or 0.2% L-arabinose (solid square) was added into the two equals, respectively. OD_600_ was measured every 30 min. The means and standard deviation of three different samples were present. For statistical analysis, two-way analysis of variance with Bonferroni post-tests were used to obtain P values for each time point: *P < 0.05; **P < 0.01; ***P < 0.001. 3 μl of serial dilutions of different cultures which were collected after induced by glucose or arabinose for 1 hour were spread onto the LA plate and incubated at 37 °C for 12 hours.

As aforementioned, the overexpression of RelE2sca could inhibit the growth of *E. coli* strain MGJ5987, a *relBE* locus defect mutant (Fig. 3c); then, we wondered whether this inhibitory effect can be neutralized by the *E. coli* antitoxin RelBeco. To explore this possibility, the antitoxin *relBeco* gene (b1564) of *E. coli* K-12 MG1655 was fused into the upstream region of *relE2sca* using SOE-PCR; subsequently, the fused fragment was cloned into the pBAD/myc-hisA vector, forming the pBAD-relBeco-relE2sca vector (supplementary Table S2). In both LB broth and LA plate, the *E. coli* MGJ5987 cells transformed with pBAD-relBeco-relE2sca, the co-expression plasmid of *relE2sca* and *relBeco*, grew better (Fig. 7a) than those transformed with the plasmid carrying *relE2sca* alone (Fig. 2b), suggesting that the *E. coli* antitoxin RelBeco could reduce the toxic effect of *S. cattleya* toxin RelE2sca. Interestingly, the toxic effect of *E. coli* toxin RelEeco (b1563) could not be abolished or alleviated by the *S. cattleya* antitoxin RelB2sca (Fig. 7b), whereas, it could be abolished by the RelB2sca mutant N61V&M68L (Fig. 7c), in which the residues Asn61 and Met68 were replaced by Val61 and Leu68, respectively. The RelB family protein has a consensus motif located at the C-terminus of their α3 helixes; the motif could be expressed as ZXnnZnnRZ, where n represents any amino acid residue, X is hydrophobic residue, and Z is hydrophobic residue with small side chain (such as Val, Ile, Leu or Ala)^14^. As revealed by the complex structure of *E. coli* RelEB, the consensus motif is critical for the interaction between RelBeco and RelEeco, via two types of interactions: salt bridge and hydrophobic interaction; the hydrophobic X and R residues are involved in the hydrophobic interactions. Formation of the RelEBeco complex will disrupt the mRNA binding and cleavage activities of RelEeco, resulting in weakened toxicity. As revealed by the sequence alignment (Fig. 1), Asn61 and Met68 of RelB2sca correspond to the X and the last Z residues of the consensus motif, respectively. Unlike the hydrophobic residues Val61 and Leu68 in RelB2eco and the RelB2sca mutant N61V&M68L, the Asn61 residue is highly hydrophilic and not suitable for the hydrophobic interaction; though Met68 is hydrophobic, it has bigger side chain compared to the Leu residue, which may further disrupt the interaction between RelE2eco and the wild type RelB2sca, leading to the complete loss of the antitoxic effect of RelB2sca.

## Discussion

*Streptomyces* usually inhabit soil and face a wide diversity of environmental conditions; the co-existence of various type II TA modules is hypothesized to favor the survival of *Streptomyces*. Based on the conserved domains of the RelBE pairs, eight putative *relBE* TA loci were identified on seven completely sequenced *Streptomyces* chromosomes (supplementary Fig. S5), including two loci in *S. cattleya*. The sequence similarities between the *S. cattleya* RelBE2sca pair and the RelBE pairs of other *Streptomyces* are very high (≥86% BLASTp identities); whereas, the similarity between *S. cattleya* RelBE2sca and RelBE1sca is relatively low (≤44% BLASTp identities). Recently, the *S. lividans yefM-yoeB* TA system has been characterized^19^; it was successfully used as the replacement of the antibiotic resistance gene, and served as a selection marker in the development of a stable plasmid expression system in *Streptomyces*^27^. The sequence similarity between the *yefM-yoeB* pair and the *relBE* modules is very low. Phylogenetic tree reconstructions also showed two distinct clades existing between the YoeB homologs and the RelE homologs in *Streptomyces* (supplementary Fig. S6). Remarkably, none *relBE* TA locus was found in the commonly used *Streptomyces* model strains, such as *S. coelicolor* A3(2), *S. lividans* TK24 or *S. avermitilis* MA-4680. Therefore, the *S. cattleya relBE2sca* TA pair identified in this study may be useful for developing a genetic tool to select and stabilize the plasmids, which can produce heterologous proteins in the common hosts.

As a paradigm TA system, the *relBE* locus has been found to be widespread. We thus examined whether the two putative *relBE* pairs predicted on the *S. cattleya* chromosome worked as functional TA modules via toxin hetero-expression and by reverse-transcriptase PCR assay. Overproduction of *S. cattleya* toxin RelE2sca resulted in the severe growth inhibition of *E. coli* MGJ5987 (MG1655 ∆10 TA) and *S. lividans* TK24, which were abolished by the co-expression of the cognate antitoxin RelB2sca. The *E. coli* RelE toxin cleaved the mRNAs displaying codon-specific manner, which cleaved the UAG faster than the other two stop codon UAA and UGA (UAG > UAA > UGA)^28^. It was also shown to specifically cleave the mRNA in the A site of the eukaryote ribosome^29^. The cleavage specificity of RelE2sca is under examination. In addition, reverse-transcriptase PCR assay showed that *relBE2sca* was transcribed as bicistronic mRNA. By using the *xylE* reporter system, the *relBE2sca* transcription was found to be inhibited efficiently by the overexpression of the RelBE2sca complex or the antitoxin RelB2sca. Subsequently, the EMSA and DNase I footprinting analysis suggested that the RelBE2sca complex repressed the transcription of *relBE2* operon by interacting with the 14-bp DNA region of the *relBE2sca* promoter. These *in silico* analyses and experimental evidence supported that the *relBE2sca* locus on the *S. cattleya* chromosome worked as a functional *relBE* system of *Streptomyces*.

The biological significance of chromosomal RelBE systems in environmental stress response has been studied in several bacteria, especially in *E. coli*^5^. The analysis of the synonymous codon usage bias showed that the Codon Adaption Index (CAI) of *relE2sca* and *relB2sca* is 0.53 and 0.43, respectively. This indicated that the expression level of the *relBE2sca* module would be expected to be lower than those of the highly expressed genes, including those coding for translation elongation factors and the ribosomal proteins (supplementary Fig. S7). We found that the functional *relBE2sca* module of *S. cattleya* was induced by osmotic pressure (Fig. 5) and nutrient limitations such as amino acid and glucose starvation (supplementary Fig. S4). Further sequence analysis showed that the expression levels of the four ClpP proteinases were also increased significantly under the osmotic pressure. The antitoxin RelB was reported to be degraded by Lon proteinase but not ClpP proteinase in *E. coli*. There was only one heat-shock Lon family proteinase found to be encoded by *S. cattleya* DSM46488 genome while six ClpP proteinases found. In this study, the antitoxin RelB2sca was revealed to be degraded by the ATP-dependent ClpP proteinases in *Streptomyces*. However, there was much work to elucidate how ClpP proteinases degrade antitoxin under diverse environmental stresses.

All the above results suggested that *relBE2sca* functions in a mode similar to that of RelBE in *E. coli*. Besides *relBE2sca*, *S. cattleya* DSM46488 also codes for another putative *relBE* locus, *relBE1sca*. The reverse-transcriptase PCR assay failed to detect the *relBE1sca* pair transcription under the condition studied (Fig. 2b); interestingly, overexpression of the toxin RelE1sca had no toxic effect to *E. coli* MGJ5987 or *S. lividans* Tk24 (Fig. 3b). As revealed by the structures of RelEeco^11^,^30^ and the sequence alignment (Fig. 1), four arginine residues (including Arg54, Arg56, Arg61, and Arg81) and one tyrosine residue (Tyr85) might be important for the mRNA binding and cleavage activities of RelE2sca. Tyr85 of RelE2sca correspond to the Tyr87 in RelE2eco, which is the catalytic residue and functions as a general acid. In RelE1sca, there is a histidine residue, His63, located at the position corresponding to the catalytic Tyr residues of RelE2sca and RelE2eco. As revealed by the studies of RNase T1 and YoeB (which is also a toxin protein), the histidine residue can also catalyze the mRNA cleavage reaction, but through a mechanism different from the tyrosine residue^10^,^11^. The wild type RelE2sca is toxic; the substitution of the Tyr85 by His85 in RelE2sca resulted in the loss of toxicity (supplementary Fig. S8a), suggesting that, like RelE2eco, RelE2sca requires a Tyr residue for catalysis. Interestingly, neither the wildtype RelE1sca nor the H63Y mutant (in which the His63 was replaced by Tyr63) is toxic to *E. coli* (supplementary Fig. S8b). Compared to the sequences of RelE2sca and RelE2eco, RelE1sca is shorter by 24 and 23 amino acids at the N-terminus, respectively. In the RelE2eco structures, the N-terminal is composed of one β-strand (β1) and one α-helix (α1); the former forms parallel β-sheet with the last β-strand (β4), whereas the later interacts with the last helix (α3), via the formation of a strong salt bridge. Lack of these interactions may affect the overall folding of RelE1sca, especially the orientations of the last helix holding the catalytic residue, leading to the complete loss of the toxin activity. Two homologous *relB-parE* systems were also predicted in the *Sinorhizobium melioti* megaplasmid pSyme^31^. The first *relB-parE* appeared to be a functional TA system while the second pair was not; however, the RelB of the second TA system retained the antitoxin activity. Interestingly, the co-expression of the putative antitoxin RelB1sca of *S. cattleya* DSM46488 could alleviate slightly the *E. coli* MGJ5987 cell growth inhibition that was caused by the toxin RelE2sca (supplementary Fig. S9). But RelB1sca exhibited a lower degree of antitoxin activity to RelE2sca, compared to that of RelB2sca or the antitoxin RelBeco of *E. coli* (Fig. 7a). Therefore, it was interesting to explore deeply why the RelE1sca was not active under condition studied, which might be helpful to understand the *relBE* family evolution.

The toxins and their cognate antitoxins appeared to interact with high specificity. There is no toxin-antitoxin cross-interaction reported between various members of the same TA families, such as *mazEF* and *chpAB* of *E. coli*^32^, *yefM-yoeB* of *Stahylococcus equorum*^33^, *parE-parD* of *Caulobacter crosotus*^34^, *paaA–parE* of *E. coli* O157:H7^35^ and *vapBC* of *Mycobacteria tuberculosis*^36^. However, the functional interaction between the *ccdAB* family had been detected in *E. coli* O157:H7^37^. Recent research showed that the toxicity of YoeB of *Streptococcus suis* could be alleviated by the non-cognate antitoxin YefM of *Streptococcus pneumoniae* but not by YefM of *E. coli*^38^. In this study, we found that the toxic effect of *S. cattleya* RelE2sca was counteracted by co-expression of *E. coli* antitoxin RelBeco (Fig. 7a) while the toxicity of RelEeco was also abolished by the mutant RelB2sca(N61V&M68L) (Fig. 7c). It will be helpful to explore the *relBE* family evolution with this new finding of the cross-complementation between the toxin RelE and the heterologous antitoxin RelB from *S. cattleya* and *E. coli*, two organisms with significant evolutionary distance and important differences in their lifestyle and the chromosome structures.

In summary, here we reported a new functional RelBE TA system *relBE2sca* found on the *S. cattleya* chromosome. Overexpression of the toxin RelE2sca caused severe growth inhibition of *E. coli ΔrelBE* mutant and *S. lividans*, and the toxicity of RelE2sca could be abolished by the co-expression of RelB2sca. More interestingly, homologous toxin-antitoxin protein interactions across the species were also found; that is, the *E. coli* antitoxin RelBeco could alleviate the toxicity of *S. cattleya* RelE2sca, and the mutant RelB2sca(N61V&M68L) but not the *S. cattleya* wild type RelB2sca could alleviate the toxicity of *E. coli* RelEeco as well. It may be helpful to investigate the important regulatory role of TA loci in *Streptomyces* physiology, environmental stress responses, and complex secondary metabolisms such as producing fluoroacetate and 4-fluorothreonine.

## Methods

### Bacterial strains and growth conditions

Strains, plasmids, and oligonucleotides used in this study were listed in the supplementary Tables S1 and S2, respectively. *E. coli* strains were grown in LB broth medium (10 g tryptone, 5 g yeast extract and 5 g NaCl per one liter) at 37 °C and *Streptomyces* strains were grown in SFM medium at 30 °C, respectively, supplemented with 100 μg/ml ampicillin, 50 μg/ml apramycin, 50 μg/ml spectinomycin, 50 μg/ml kanamycin or 25 μg/ml thiostrepton as required. Conjugative transfer of DNA from *E. coli* ET12567/pUZ8002 to *Streptomyces* was performed as described by Kieser *et al.*^39^ The same amount of donor cells and recipient cells were used in each individual conjugation.

### *in silico* identification of Type II TA loci in *S. cattleya* DSM46488

Nucleotide sequences and annotations of the completely sequenced *S. cattleya* DSM46488 chromosome and plasmid were downloaded from the NCBI RefSeq Project under the accession of NC_017586 and NC_017585, respectively. The toxin or antitoxin protein homologs were firstly obtained by HMMer-based conserved domain searches with an e-value cut-off of 0.01. The 343 hidden Markov model (HMM) profile profiles had been built from the Type II TA proteins archived by TADB. Then, the short homologs with the size of 30 - 500 a.a. were kept as TA candidates. Finally, two flanking TA candidate genes with the intergenic distances of −20 to 30 bp were paired as an operon structure, and thus predicted as a putative Type II TA locus in *S. cattleya* DSM46488 (supplementary Table S1).

### RNA isolation, RT-PCR, and quantitative real-time quantitative PCR analysis

Total RNA samples were extracted from *S. cattleya* DSM46488 culturing in TSBY for 2–3 days by using Redzol reagent and the contaminated genomic DNA was eliminated by treating with RNase-free DNase I. RNA concentrations and integrity were determined by Nanodrop and agarose gel electrophoresis, respectively. Reverse transcriptase PCR (RT-PCR) was carried out using a QuantiTect Reverse Transcription Kit according to the manufacturer’s protocol. For the co-transcription assay, the genes specific primers were used for RT-PCR analysis. In addition, to assess the transcription of *relB2sca* and the *clpP* proteinases responding to the osmotic stress, *S. cattleya* DSM46488 was grown in TSBY medium for 2–3 days and then inoculated to the fresh minimal medium with 1:10 amount. After the cells were grown to log phase, 0.5 M sucrose and 0.5 M NaCl (final concentration) were added into the medium and the cells were kept to grow for 3 hours. Finally, total RNA was isolated as described above. Primers targeting genes *relB2sca*, *clpP1*, *clpP2*, *clpP3*, *clpP4*, *clpP5 *and *clpP6* were designed for detecting the mRNA expression (supplementary Table S3). Real-time quantitative PCR was carried out with an ABI 7500 fast system. Calculations were performed by using 16S rRNA as an internal standard. The 2^−∆∆CT^ method was used to determine the relative gene expression^40^. A fold change of >2 or <−0.5 between the treated and untreated cells was considered a significant difference.

### Plasmid construction

Plasmids used in this study (supplementary Table S2) were constructed as the following methods by using the primers listed in the supplementary Table S3.

(i) pBAD-relE1sca, pBAD-relE2sca and pBAD-relBE2sca. Primers were used to amplify the *relE1sca*, *relE2sca* and the intact *relBE2sca* operon from the *S. cattleya* DSM46488 genomic DNA (*S. cattleya* gDNA), respectively. The PCR products were digested with *Nco*I and *Xho*I enzymes, which were then cloned into pBAD-myc/hisA, resulting in plasmids pBAD-relE1sca, pBAD- relE2sca and pBAD-relBE2sca.

(ii) pIB139-relE1sca, pIB139-relB1sca, pIB139-relE2sca and pIB139-relB2sca. The toxin gene *relE1sca* was amplified with primers IBrelE1F and IBrelE1R from the *S. cattleya* gDNA and then cloned into pIB139, generating pIB139-relE1sca. Similar strategy was employed to obtain pIB139-relB1sca, pIB139-relE2sca and pIB139-relB2sca.

(iii) pACYC-relB2sca-relE2scahis. The toxin gene *relE2sca* was amplified with primers E2exF and E2exR from the *S. cattleya* gDNA. The PCR product was treated with *Bam*HI and *Hin*dIII enzymes and then cloned into the MCS1 of pACYCDuet1, generating plasmid pACYC-relE2scahis, which expressed the toxin protein with a six-histidine tag at the N-terminal. The PCR product was also digested with *Nde*I and *Xho*I and then cloned into the MCS2 of pACYC-relE2scahis, resulting in plasmid pACYC-relB2sca-relE2scahis.

(iv) pRSF-ClpP1X, pRSF-ClpP2X, pRSF-ClpP3X, pRSF-ClpP4X, pRSF-ClpP5X and pRSF-ClpP6X. The proteinase chaperone gene *clpX* was amplified from the *S. cattleya* gDNA with the primers clpXF and clpXR and then cloned into the MCS1 of pRSFDuet1 with *Nco*I and *Hin*dIII, producing pRSFDuet1-clpX. The proteinase gene *clpP1* was amplified from the *S. cattleya* gDNA with primers clpP1F and clpP1R and then cloned into the MCS2 of pRSFDuet-clpX, producing plasmid pRSF-ClpP1X. The plasmids pRSF-ClpP2X, pRSF-ClpP3X, pRSF-ClpP4X, pRSF-ClpP5X and pRSF-ClpP6X were obtained with the same strategy.

### Toxicity evaluation in *E. coli* in a liquid and solid medium

*E. coli* MGJ5987, a ten-TA locus defect mutant (*ΔchpB, ΔrelBE, ΔdinJ-yafQ, ΔyefM-yoeB, ΔhigBA, ΔprlF-yhaV, ΔyafNO, ΔmqsRA, ΔhicAB*), transformed with corresponding plasmids was grown in LB broth supplemented with 100 μg/ml ampicillin at 37 °C. When the OD_600_ reached to about 0.2, the cultures were divided into two equal parts. The one was grown in the presence of 0.2% glucose (inhibition of the promoter P_BAD_) while the other was grown in the presence of 0.2% L-Arabinose (induction of the promoter P_BAD_). OD_600_ was measured every 30 minutes to monitor the *E. coli* cell growth. In addition, the samples were also obtained after induction for 1 h, and 5 μl drops of different dilutions were spread onto the LA plate supplemented with 100 μg/ml ampicillin. The LA plates were incubated overnight at 37 °C.

### Purification of RelE2sca-His_6_, RelBsca and RelBE2sca-His_6_

RelBE2sca-His_6_ complex was overproduced in *E. coli* BL21(DE3) transformed with pACYC-relB2sca-relE2scahis (supplementary Table S2). After induced by 0.5 mM IPTG at 37 °C for 4 hours, cells were harvested by centrifugation at 8,000 g for 10 minutes at 4 °C. The cell pellet was re-suspended in lysis buffer (25 mM Tris-HCl, pH7.4, 500 mM NaCl, 50 mM imidazole) and then sonicated and centrifuged by centrifugation at 10,000 g for 45 minutes at 4 °C to remove cell debris. The supernatant was applied to a column containing 2 ml of NTA-Ni resin. The column was washed three times with 10 ml of the buffer A (25 mM Tris-HCl, pH7.4, 500 mM NaCl, 50 mM imidazole). RelBE2sca-His_6_ complex was eluted with 5 ml of the buffer B (25 mM Tris-HCl, pH7.4, 500 mM NaCl, 500 mM imidazole). The overexpression of RelE2sca-His_6_ and RelB2sca was done as the same as the expression of RelBE2sca-His_6_. And the protocol described by Sterckx *et al.*^41^ was employed to purify the RelE2sca-His_6_ and RelB2sca from the His6-RelBE2sca complex. The purified RelBE2sca-His_6_ was finally subjected to size exclusion chromatography analysis.

### Electrophoretic mobility shift assay

DNA probe was amplified by using PCR with FAM-labeled oligonucleotide primers (supplementary Table S3) from the *S. cattleya* gDNA and purified with a nucleic acid purification kit. The labeled DNA probe was incubated with different amounts of proteins in EMSA buffer (50 mM Tris-HCl, pH 7.5; 10 mM MgCl_2_; 1 mM DTT and 100 mM NaCl) at room temperature for 30 minutes. The mixtures were then subjected to 6% native polyacrylamide gel electrophoresis at 100 V for 1 hour at 4 °C. Unlabeled DNA probe used as a specific competitor was amplified and purified as described previously. In the competitive experiments, the labeled and unlabeled DNA probes in different ratio were mixed with the same amount of proteins in EMSA buffer, then incubated at room temperature for 30 minutes and separated by 6% native polyacrylamide gel electrophoresis. The images of gels were obtained by using Bio-Rad Molecular Imager Gel Doc XR+ System.

### DNase I footprinting assay

The 5′-FAM-labeled DNA probes and the reaction system were the same as the EMSA experiments. The mixtures were treated with DNase I (0.25 units, ThermoFisher Fermentas) at room temperature for 10 minutes. The reaction was stopped by adding 0.25 μl 0.5 M EDTA and incubating in a water bath at 75 °C for 15 minutes. The treated DNA fragments were purified with the nucleic acid purification kit and eluted with 50 μl double distilled water. The purified DNA fragments were mixed with HiDi formamide and GeneScan-500 LIZ size standards, assayed with Applied Biosystems 3730XL DNA analyzer (manufactured by the Jieli Company, Shanghai). Electropherograms were analyzed using the GENEMAPPER software (Applied Biosystems).

### *In vivo* degradation of RelB2sca in *E. coli*

To elucidate the degradative mechanism of RelB2sca, *E. coli* BL21(DE3)/pLysS were transformed with pBAD-relB2scahis and pRSFDuet-1, pBAD-relB2scahis and pRSF-ClpP1X, pBAD-relB2scahis and pRSF-ClpP2X, pBAD-relB2scahis and pRSF-ClpP3X, pBAD-relB2scahis and pRSF-ClpP4X, pBAD-relB2scahis and pRSF-ClpP5X, pBAD-relB2scahis and pRSF-ClpP6X, respectively. The *intracellular* stability of plasmid-encoding RelB2sca-His_6_ was measured using *in vivo* degradation experiments. *E. coli* cells transformed with the corresponding plasmids were grown at 37 °C until that the OD_600_ reached 0.5. L-arabinose was added to a final concentration of 0.2% to induce His6-relB2sca expression. After the cells induced by L-arabinose at 37 °C for 4 hours, IPTG was added to a final concentration of 0.5 mM to induce ClpP1X, ClpP2X, ClpP3X, ClpP4X, ClpP5X or ClpP6X expression for 2 hours. Then spectinomycin was added to a final concentration of 200 μg/ml to block translation. Samples (3-ml aliquots) were collected every 2 hours. Cells were pelleted by centrifugation at 4 °C and stored at −80 °C.

### Preparation of protein extracts and Western blotting

Cells were re-suspended in lysis buffer (25 mM Tris-HCl, 500 mM NaCl) and then disrupted with ultrasound according to the manufacturer’s recommendations. Lysates were centrifuged at 10,000 rpm/min for 60 minutes at 4 °C. And the 6×SDS gel loading buffer was added. Then the mixture was separated by SDS-PAGE and transferred to a poly-vinylidene difluoride membrane. RelB2sca-His_6_ proteins were detected by using an anti-His antibody. Densitometric analysis of the scanned images from the exposed film was performed with ImageJ^42^.

## Additional Information

**How to cite this article**: Li, P. *et al.* Identification and characterization of chromosomal *relBE* toxin-antitoxin locus in *Streptomyces cattleya* DSM46488. *Sci. Rep.* **6**, 32047; doi: 10.1038/srep32047 (2016).

## Supplementary Material

Supplementary Information
