# Supplementary material for: Identification and characterization of chromosomal relBE toxin-antitoxin locus in Streptomyces cattleya DSM46488
Source: Sci Rep. 2016 Aug 18;6:32047. doi: 10.1038/srep32047 (PMC4989188; doi:10.1038/srep32047)
Supplement: Supplementary Information [file srep32047-s1.doc]

**Identification and characterization of chromosomal *relBE* toxin-antitoxin locus in *Streptomyces cattleya* DSM46488**

Peng Li ^1^, Cui Tai ^1^, Zixin Deng ^1^, Jianhua Gan ^2^, Marco R. Oggioni ^3^, Hong-Yu Ou ^1,*^

1. State Key Laboratory of Microbial Metabolism, Joint International Laboratory on Metabolic & Developmental Sciences, School of Life Sciences & Biotechnology, Shanghai Jiao Tong University, Shanghai, China

^2^. Department of Physiology and Biophysics, School of Life Sciences, Fudan University, Shanghai, China

^3^. Department of Genetics, University of Leicester, Leicester, UK

*Correspondence and requests for materials should be addressed to H.Y.O (hyou@sjtu.edu.cn)

Key words: toxin-antitoxin system, RelBE, ClpP proteinase, *Streptomyces cattleya*

**Supplementary Information**

**Table S1.** Thirty-three putative type II toxin-antitoxin loci identified on the linear chromosome and mega-plasmid of *Streptomyces cattleya* DSM46488.

**Table S2.** Strains and plasmids used in this study. Km^R^, Ap^R^, Cm^R^, Thio^R^ denote kanamycin, ampicillin, chloramphenicol and thiostrepton resistance, respectively.

**Table S3.** Oligonucleotides used in this study.

**Figure S1.** Purification of RelBE2sca-His_6_ complex, RelE2sca-His_6_ and RelB2sca.

**Figure S2.** Effect of the *S. cattleya* RelBE proteins (RelE1sca, RelB1sca, RelE2sca, and RelB2sca) on the growth of wild-type *S. lividans* TK24.

**Figure S3.** DNase I footprinting assay for locating the binding sites of *S. cattleya* RelBE2sca complex into the promoter region of *relBE2sca*.

**Figure S4**. Transcription of the antitoxin gene *relB2sca* and the ClpP proteinase genes under different environmental stresses.

**Figure S5.** Alignment maps of the *relBE* type II toxin-antitoxin loci found on the completely sequenced *Streptomyces* chromosomes.

**Figure S6.** Inferred phylogenetic relationships of the 9 RelE and 2 YoeB toxin proteins of *Streptomyces* and *E. coli*.

**Figure S7.** Scatter plots of Codon Adaption Index (CAI) values against the third codon position GC content (GC3) values for the 5822 annotated genes on the *S. cattleya* DSM46488 chromosome.

**Figure S8**. Effect of over-expression of the RelE2sca Y85H mutant and RelE1sca H63Y on the growth of *E. coli* MGJ9587.

**Figure S9.** Interaction of the RelE2sca toxin and the RelB1sca antitoxin of *S. cattleya*.

**Figure S10.** Original images of cropped gels used in Fig. 2.

**Table S1.** Thirty-three putative type II toxin-antitoxin loci identified on the linear chromosome and mega-plasmid of *Streptomyces cattleya* DSM46488.

| **#** | **Product** | **Locus tag** | **Location** | **Strand** | **Length(a.a.)** | **Family/Domain** |
| --- | --- | --- | --- | --- | --- | --- |
| **Linear chromosome** | | | |  |  |  |
| 1 | Toxin | SCATT_03640 | 411752..412132 | - | 126 | Doc |
|  | Antitoxin | SCATT_03650 | 412129..412425 | - | 98 | Phd |
| 2 | Toxin | SCATT_07660 | 859198..859605 | - | 135 | COG3832 |
|  | Antitoxin | SCATT_07680 | 859602..859913 | - | 103 | ArsR |
| 3 | Toxin | SCATT_12430 | 1358627..1358836 | + | 69 | DUF397 |
|  | Antitoxin | SCATT_12420 | 1357819..1358643 | + | 274 | Xre |
| 4 | Toxin | SCATT_13740 | 1488184..1488444 | + | 86 | DUF397 |
|  | Antitoxin | SCATT_13730 | 1487303..1488187 | + | 294 | Xre |
| 5 | Toxin | SCATT_14000 | 1518854..1519075 | - | 73 | DUF397 |
|  | Antitoxin | SCATT_14010 | 1519072..1519902 | - | 276 | Xre |
| 6 | Toxin | SCATT_14440 | 1561951..1562196 | + | 81 | DUF397 |
|  | Antitoxin | SCATT_14430 | 1561121..1561954 | + | 277 | Xre |
| 7 | Toxin | SCATT_19180 | 2036402..2036617 | + | 71 | DUF397 |
|  | Antitoxin | SCATT_19170 | 2035538..2036398 | + | 286 | Xre |
| **8** | **Toxin** | **SCATT_20930** | **2238737..2238937** | **+** | **66** | **RelE1** |
|  | **Antitoxin** | **SCATT_20920** | **2238399..2238671** | **+** | **90** | **RelB1** |
| 9 | Toxin | SCATT_23210 | 2461632..2461841 | + | 69 | DUF397 |
|  | Antitoxin | SCATT_23200 | 2460566..2461423 | + | 285 | Xre |
| 10 | Toxin | SCATT_23900 | 2543012..2543248 | + | 78 | DUF397 |
|  | Antitoxin | SCATT_23890 | 2542152..2543015 | + | 287 | Xre |
| 11 | Toxin | SCATT_25240 | 2666334..2666585 | - | 83 | DUF397 |
|  | Antitoxin | SCATT_25250 | 2666582..2667391 | - | 269 | Xre |
| 12 | Toxin | SCATT_26040 | 2747365..2747832 | - | 155 | COG1246 |
|  | Antitoxin | SCATT_26050 | 2747902..2748297 | - | 131 | COG3682 |
| 13 | Toxin | SCATT_26970 | 2856185..2856595 | - | 136 | Pin |
|  | Antitoxin | SCATT_26990 | 2856592..2856822 | - | 76 | Rhh |
| 14 | Toxin | SCATT_28290 | 2968893..2970509 | + | 538 | PIN |
|  | Antitoxin | SCATT_28280 | 2968237..2968896 | + | 219 | Xre |
| 15 | Toxin | SCATT_31770 | 3353877..3354296 | + | 139 | Pin |
|  | Antitoxin | SCATT_31750 | 3353659..3353880 | + | 73 | VapB |
| 16 | Toxin | SCATT_32830 | 3481150..3481725 | - | 191 | COG2856 |
|  | Antitoxin | SCATT_32840 | 3481722..3482162 | - | 146 | Xre |
| 17 | Toxin | SCATT_33790 | 3584256..3584468 | - | 70 | DUF397 |
|  | Antitoxin | SCATT_33800 | 3584468..3585313 | - | 281 | Xre |
| 18 | Toxin | SCATT_34680 | 3696382..3696573 | + | 63 | DUF397 |
|  | Antitoxin | SCATT_34670 | 3695484..3696371 | + | 295 | Xre |
| 19 | Toxin | SCATT_37320 | 4019846..4020082 | + | 78 | DUF397 |
|  | Antitoxin | SCATT_37310 | 4019025..4019834 | + | 269 | Xre |
| **20** | **Toxin** | **SCATT_39270** | **4216503..4216766** | **-** | **87** | **RelE2** |
|  | **Antitoxin** | **SCATT_39280** | **4216759..4217028** | **-** | **89** | **RelB2** |
| 21 | Toxin | SCATT_39510 | 4245384..4245587 | + | 67 | DUF397 |
|  | Antitoxin | SCATT_39500 | 4244567..4245373 | + | 268 | Xre |
| 22 | Toxin | SCATT_41380 | 4441302..4441505 | + | 67 | DUF397 |
|  | Antitoxin | SCATT_41370 | 4440416..4441291 | + | 291 | Xre |
| 23 | Toxin | SCATT_42840 | 4602198..4602656 | - | 152 | Pin |
|  | Antitoxin | SCATT_42850 | 4602646..4602915 | - | 89 | Phd |
| 24 | Toxin | SCATT_43000 | 4619015..4619221 | - | 68 | pfam04149 |
|  | Antitoxin | SCATT_43010 | 4619233..4620186 | - | 317 | cd00093 |
| 25 | Toxin | SCATT_43290 | 4655273..4655476 | - | 67 | DUF397 |
|  | Antitoxin | SCATT_43310 | 4655489..4655785 | - | 98 | Xre |
| 26 | Toxin | SCATT_43710 | 4699849..4700109 | - | 86 | DUF397 |
|  | Antitoxin | SCATT_43730 | 4700106..4700951 | - | 281 | Xre |
| 27 | Toxin | SCATT_49490 | 5349869..5350129 | + | 86 | DUF397 |
|  | Antitoxin | SCATT_49470 | 5349027..5349863 | + | 278 | Xre |
| 28 | Toxin | SCATT_51080 | 5520067..5520255 | - | 62 | DUF397 |
|  | Antitoxin | SCATT_51090 | 5520252..5521109 | - | 285 | Xre |
| 29 | Toxin | SCATT_52210 | 5643247..5643462 | - | 71 | DUF397 |
|  | Antitoxin | SCATT_52200 | 5642392..5643132 | - | 246 | HEPN |
| 30 | Toxin | SCATT_55620 | 5976124..5976387 | + | 87 | DUF397 |
|  | Antitoxin | SCATT_55630 | 5976527..5977429 | + | 300 | Xre |
| **Linear mega-plasmid** | | | |  |  |  |
| 31 | Toxin | SCATT_p04360 | 436654..437172 | + | 172 | COG2856 |
|  | Antitoxin | SCATT_p04350 | 436127..436642 | + | 171 | Xre |
| 32 | Toxin | SCATT_p06640 | 660021..660230 | + | 69 | DUF397 |
|  | Antitoxin | SCATT_p06630 | 659188..660024 | + | 278 | Xre |
| 33 | Toxin | SCATT_p14790 | 1538211..1538738 | - | 175 | COG2856 |
|  | Antitoxin | SCATT_p14800 | 1538775..1539227 | - | 150 | Xre |

**Table S2.** Strains and plasmids used in this study. Km^R^, Ap^R^, Cm^R^, Thio^R^ denote kanamycin, ampicillin, chloramphenicol and thiostrepton resistance, respectively.

| **Strain or Plasmid** | **Description** | **Source or reference** |
| --- | --- | --- |
| *E. coli* K-12 MG1655 | *E. coli* K-12 wild type | Lab collection |
| *E. coli* MGJ5987 (Δ10TA) | MG1655 *ΔmazF* ΔchpB ΔrelBE Δ(dinJ-yafQ) Δ(yefM-yoeB) ΔhigBA*  *Δ(prlF-yhaV) ΔyafNO ΔmqsRA ΔhicAB* | [^1^](#_ENREF_1) |
| *E. coli* DH10B | F−, *φ80lacZ, M15, endA, recA, hsdR(rk−mk−), supE, thi, gyrA, relA,* Δ*(lac*ZYA*-argF)U169* | GIBCO BRL |
| *E. coli* BL21(DE3) | F^–^, *ompT, gal, dcm, lon, hsdS_B_, (rB^-^ mB^-^)* λ(DE3) | Novagene |
| *E. coli* BL21(DE3)pLysS | F^–^ *ompT gal dcm lon hsdS_B_(rB^-^ mB^-^)* λ(DE3) pLysS(cm^R^) | Novagene |
| ET12567/pUZ8002 | *Dam 13::Tn9 dcm-6 hsdM hsdR, oriT-RP4, host strain for conjugation* | [^2^](#_ENREF_2) |
| *S. lividans* TK24 | *str-6*, SLP2^-^, SLP3^-^ | [^3^](#_ENREF_3) |
| *S. cattleya* DSM46488 | Wild type | [^4^](#_ENREF_4) |
| pBAD/myc-hisA | pBR322, *Ap^R^*, *araC*, pBAD | Life Tech. |
| pBAD-relE1sca | pBAD/myc-hisA containing *relE1sca* gene | This work |
| pBAD-relE2sca | pBAD/myc-hisA containing *relE2sca* gene | This work |
| pBAD-relBE2sca | pBAD/myc-hisA containing intact *relBE2sca* operon | This work |
| pBAD-relB1sca-relE2sca | pBAD/myc-hisA containing *relB1sca* and *relE2sca* genes | This work |
| pBAD-relBeco-relE2sca | pBAD/myc-hisA containing *relBeco* and *relE2sca* genes | This work |
| pBAD-relB2sca-relEeco | pBAD/myc-hisA containing *relB2sca* and *relEeco* genes | This work |
| pBAD-relB2(N55V&M62L)-b1563 | pBAD/myc-hisA containing the *relB2sca(N55V&M62L)* and *relBeco* genes | This work |
| pBAD-relB2scahis | pBAD/myc-hisA containing *relB2sca* gene | This work |
| pACYCDuet-1 | p15A, *Cm*^R^, *lacI*, T7 | Novagen |
| pACYC-relB2sca-relE2scahis | pACYCDuet-1 containing *relB2sca* and *relE2sca* genes | This work |
| pRSFDuet-1 | p15A, *Km*^R^, *lacI*, T7 | Novagen |
| pRSF-ClpP1X | pRSFDuet-1 containing *clpP1* and *clpX* genes | This work |
| pRSF-ClpP2X | pRSFDuet-1 containing *clpP1* and *clpX* genes | This work |
| pRSF-ClpP3X | pRSFDuet-1 containing *clpP1* and *clpX* genes | This work |
| pRSF-ClpP4X | pRSFDuet-1 containing *clpP1* and *clpX* genes | This work |
| pRSF-ClpP5X | pRSFDuet-1 containing *clpP1* and *clpX* genes | This work |
| pRSF-ClpP6X | pRSFDuet-1 containing *clpP1* and *clpX* genes | This work |
| pIB139 | Derivative of pSET152, containing *PermE, oriT, attP, φC31 int, and aac(3)IV* | [^5^](#_ENREF_5) |
| pIB139-relB1sca | pIB139 containing *relB1sca* gene | This work |
| pIB139-relB2sca | pIB139 containing *relB2sca* gene | This work |
| pIB139-relE1sca | pIB139 containing *relE1sca* gene | This work |
| pIB139-relE2sca | pIB139 containing *relE2sca* gene | This work |
| pIB139-relBE2sca | pIB139 containing *relB2sca* and *relE2sca* genes | This work |
| pJTU1278 | ColEI, pIJ101, *oriT, Ap^R^, thio^R^* | [^6^](#_ENREF_6) |
| pJTU1278-PermE-relB2sca | pJTU1278 containing strong constitutive promoter ermE and *relB2sca* gene | This work |
| pJTU1278-PermE-relBE2sca | pJTU1278 containing strong constitutive promoter ermE and the intact relBE2sca operon | This work |
| pJTU3700 | Derivative of pSET152 containing *oriT, attP, φC31 int, and aac(3)IV* and the promoter-less reporter gene *xylE* | Dai et al. unpublished |
| pJTU3700-PrelBE2sca | pJTU3700 containing the promoter of *relBE2sca* module | This work |

**Table S3.** Oligonucleotides used in this study. F indicates the forward primer and R indicates the reverse primer. All restriction enzyme sites are underlined. FAM indicates that the oligonucleotide is FAM labelled at the 5’ end.

| Primer name | Sequence (5’-3’) | |
| --- | --- | --- |
| Detection of the genetic organization | | |
| 16S_rtF1 | CTGGCGGCGTGCTTAACACA | |
| 16S_rtR1 | CCCACGTGTTACTCACCCGT | |
| relB1_rtF | CACTCCGACGGTCATCACG | |
| relB1_rtR | CTCCGTCATCGTCACGGTC | |
| relE1_rtF | CATCCTGACCGCACTGACC | |
| relE1_rtR | ACTTGACGACGAGGATGACG | |
| relB2_rtF | GACGTGCCCACGGTGAT | |
| relB2_rtR | GCGTCTCCCTCATGATGTCC | |
| relE2_rtF | GACATGGCTCTCCGCATTCT | |
| relE2_rtR | ACCACCAGCTCACCATTGTC | |
| relBE1_rtF | TTCGAAGCGCTGGAGGAAG | |
| relBE1_rtF | ACTTGACGACGAGGATGACG | |
| relBE2_rtF | GTGGTGTCCATCGAAGTGCT | |
| relBE2_rtF | ACCACCAGCTCACCATTGTC | |
| Evaluation of *S. cattleya* toxins in *E. coli* | | |
| E1F1 | GGGCCATGGTGCGCATCCTGACCGCA | |
| E1R1 | GGGCTCGAGTCACAGGTTGCGGTGGAC | |
| E2F1 | GGGCCATGGTGAGTGAGTACCGCACCG | |
| E2R1 | GGGCTCGAGTCAGGCGCCGTAGACGGTTG | |
| BE1F1 | GGGCTCGAGAATGACACAACCGCTGCCC | |
| BE1R1 | CCCAAGCTTTCAGGCGCCGTAGACGGTTGxho | |
| Over-expression of RelB2 and RelE2 | | |
| B2exF | GGGCATATGACACAACCGCTGCCC | |
| B2exR | CCCAAGCTTTCAGGCGCCGTAGACGGT | |
| E2exF | CCCGGATCCGGTGAGTGAGTACCGCACC | |
| E2exR | CCCAAGCTTTCAGGCGCCGTAGACGGT | |
| Auto-regulation of *relBE2* module | | |
| promoterF | GCCTACCAACCTCTGCCG |  |
| promoterR | GCGCTCAAGAGTACCGGG |  |
| ermE-relB2F | CCCGAATTCGGCTGCAGGTCGACTCTAGT |  |
| ermE-relB2R | CCCACTAGTTCACTCACTGCGCGCCAGC |  |
| ermE-relBE2R | GGGACTAGTTCAGGCGCCGTAGACGGTT |  |
| EMSA_F | GCCTACCAACCTCTGCCG |  |
| EMSA_R | FAM-GCGCTCAAGAGTACCGGG |  |
| P2MF | ACAACGGTTGTTGTCGCGC |  |
| P2MR | GACAACAACCGTTGTCCCGGTACTCTTGAGCGCA |  |
| relB2-b1563T-F1 | GGGCCATGGATATGGAGTCCATCCGCGATGT |  |
| relB2-b1563T-R1 | TCACTCACTGCGCGCCAGC |  |
| relB2-b1563T-F2 | CGCTGGCGCGCAGTGAGTGATGGCGTATTTTCTGGATT |  |
| relB2-b1563T-R2 | GGGCTCGAGTCAGAGAATGCGTTTGACCG |  |
| b1564A-relE2-F1 | GGGCCATGGATGGGTAGCATTAACCTGC |  |
| b1564A-relE2-R1 | ACGGTGCGGTACTCACTCACTCAGAGTTCATCCAGCGTCA |  |
| b1564A-relE2-F2 | GTGAGTGAGTACCGCACCGT |  |
| b1564A_relE2-R2 | CCCAAGCTTTCAGGCGCCGTAGACGGTTG |  |
| relB1-relE2-F1 | GGGCCATGGATATGTCCGAGAACACCGTGA |  |
| relB1-relE2-R1 | ACGGTGCGGTACTCACTCACTTACGCGGTGTCACCGTCG |  |
| relB1-relE2-F2 | GTGAGTGAGTACCGCACCGT |  |
| relB1-relE2-R2 | GGGCTCGAGTCAGGCGCCGTAGACGGTTG |  |
| qRT-PCR | | |
| clpP1qF | ATGGAGGCCCGCTACATCGT | |
| clpP1qR | TGCACGCCGAGGAAGATCAC | |
| clpP2qF | CTCGGTGACGCGGTCTACAA | |
| clpP2qR | AGATCCGGTTGGCGATGT | |
| clpP3qF | AGCTGGTGCACCTCGAATAC | |
| clpP3qR | TGTACCGCATGGTGTCGTAG | |
| clpP4qF | AAGATCCTCATGCACCAGCC | |
| clpP4qR | CTCGGCCATCTCCTTCTTGG | |
| clpP5qF | ATCCAGACGGTGTGCATAGG | |
| clpP5qR | ATGAGGATCTTGGCGTTGGG | |
| clpP6qF | GTGCCTTCCAGTTCCCCC | |
| clpP6qR | TCTTCGGTGTCGAAGTGCTC | |
| lonqF | CGAACTGGTCCGGGAGTACA | |
| lonqR | AGACGTCGTCGATCTGCTG | |
| Western blotting analysis | | |
| pBAD-relB2HisF | GGGCTCGAGAATGCATCATCACCATCACCACACACAACCG  CTGCCCATG | |
| pBAD-relB2HisR | CCCAAGCTTTCACTCACTGCGCGCCAGCGT | |
| clpXF | GGGCCATGGGCGTGGCACGCATCGGTGAC | |
| clpXR | GGGAAGCTTCTACGCGCTCTTCTCGTG | |
| clpP1F | GGGCATATGAACACCCGCAACCCG | |
| clpP1R | GGGCTCGAGTCAGGCCGAAAGCGCG | |
| clpP2F | GGGCATATGACGACTCCGCAGATCG | |
| clpP2R | GGGCTCGAGTCAGGCACCGGTACCGC | |
| clpP3F | GGGCATATGCGCCAGCCGTCCGCAG | |
| clpP3R | GGGCTCGAGTCACCTCGGGCCGGGTGCG | |
| clpP4F | GGGCATATGGCTTACCAGGACGGCCG | |
| clpP4R | GGGCTCGAGTCAGTCCGTCTTGGTGCCGC | |
| clpP5F | GGGCATATGGCACCGCACTCGCACC | |
| clpP5R | GGGCTCGAGTCAGACATGGCGCCCGGCG | |
| clpP6F | GGGCATATGTTCGACCGATTCACCGA | |
| clpP6R | GGGCTCGAGCTACGAGGCGCCGGACCC | |
| Evaluation of the *S. cattleya* toxin in *S. lividans* TK24 | | |
| IBrelE1F | GGGCATATGCGCATCCTGACCGCA | |
| IBrelE1R | GGGTCTAGATCACAGGTTGCGGTGGACG | |
| IBrelB1F | GGGCATATGTCCGAGAACACCGTGAC | |
| IBrelB1R | GGGTCTAGATTACGCGGTGTCACCGTCG | |
| IBrelE2F | GGGCATATGAGTGAGTACCGCACCG | |
| IBrelE2R | GGGTCTAGATCAGGCGCCGTAGACGGT | |
| IBrelB2F | GGGCATATGACACAACCGCTGCCCA | |
| IBrelB2R | GGGTCTAGATCACTCACTGCGCGCCAG | |


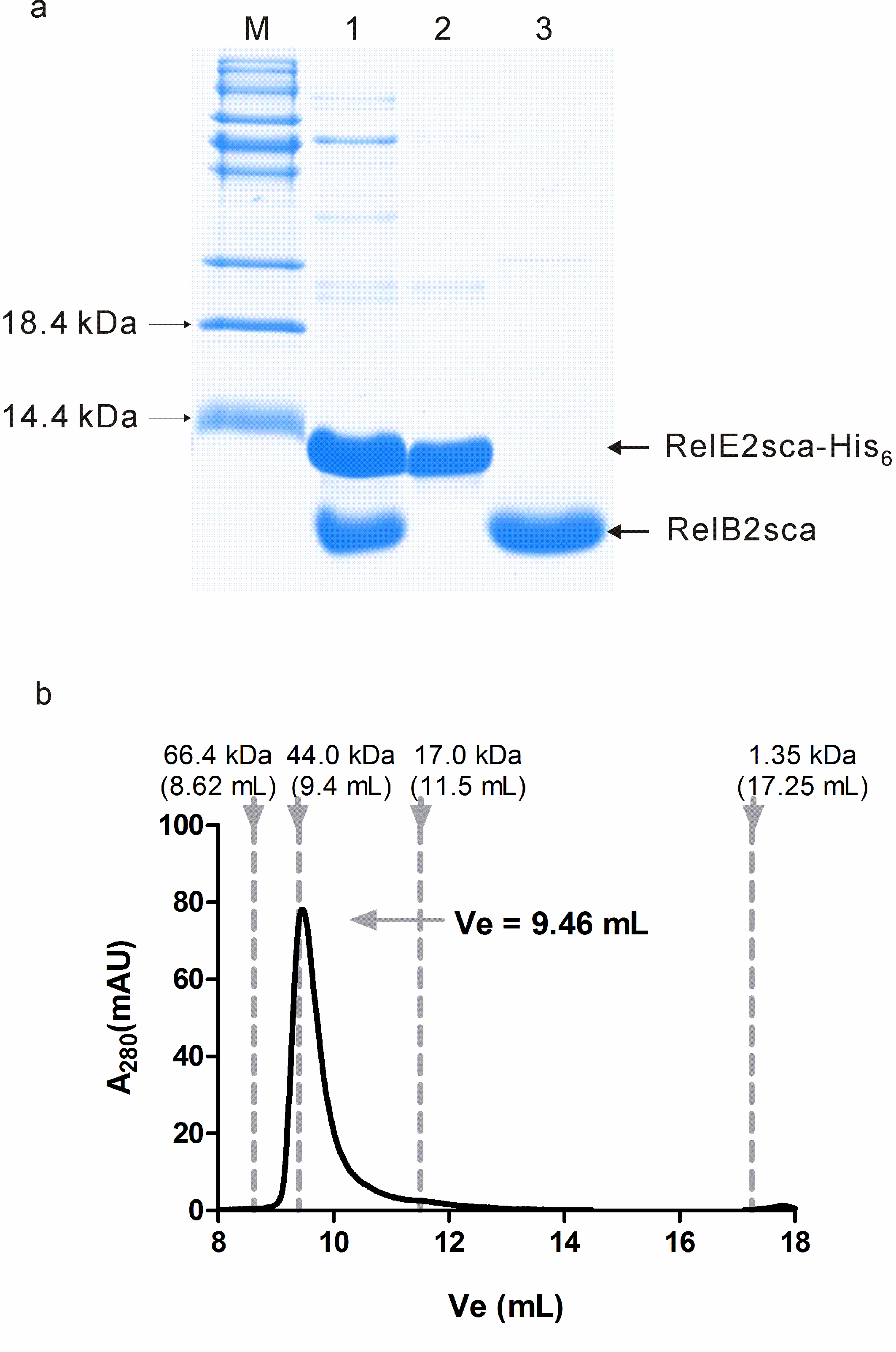


**Supplementary Figure S1.** Purification of RelBE2sca-His_6_ complex, RelE2sca-His_6_ and RelB2sca. (a) RelBE2sca-His_6_ complex was purified by nickel affinity chromatography. RelE2sca-His_6_ and RelB2sca were purified from the purified RelBE2sca-His_6_ complex under denatured conditions and refolded in dialysis buffer. Lane M indicated the proteins standards. Lane 1, 2 and 3 indicated the RelBE2sca-His_6_ complex, RelE2sca-His_6_ (11.54 kDa) and RelB2 (10.32 kDa), respectively. (b) Size exclusion chromatography of purified RelBE2sca-His_6_ complex. The elution volumes of standard proteins (BSA, 66.4 kDa; ovalbumin, 44.0 kDa; myoglobin, 17.0 kDa and vitamin B-12, 1.35 kDa) were indicated by the dash lines. The Ve was the elution volume of the purified RelBE2sca-His_6_ complex.


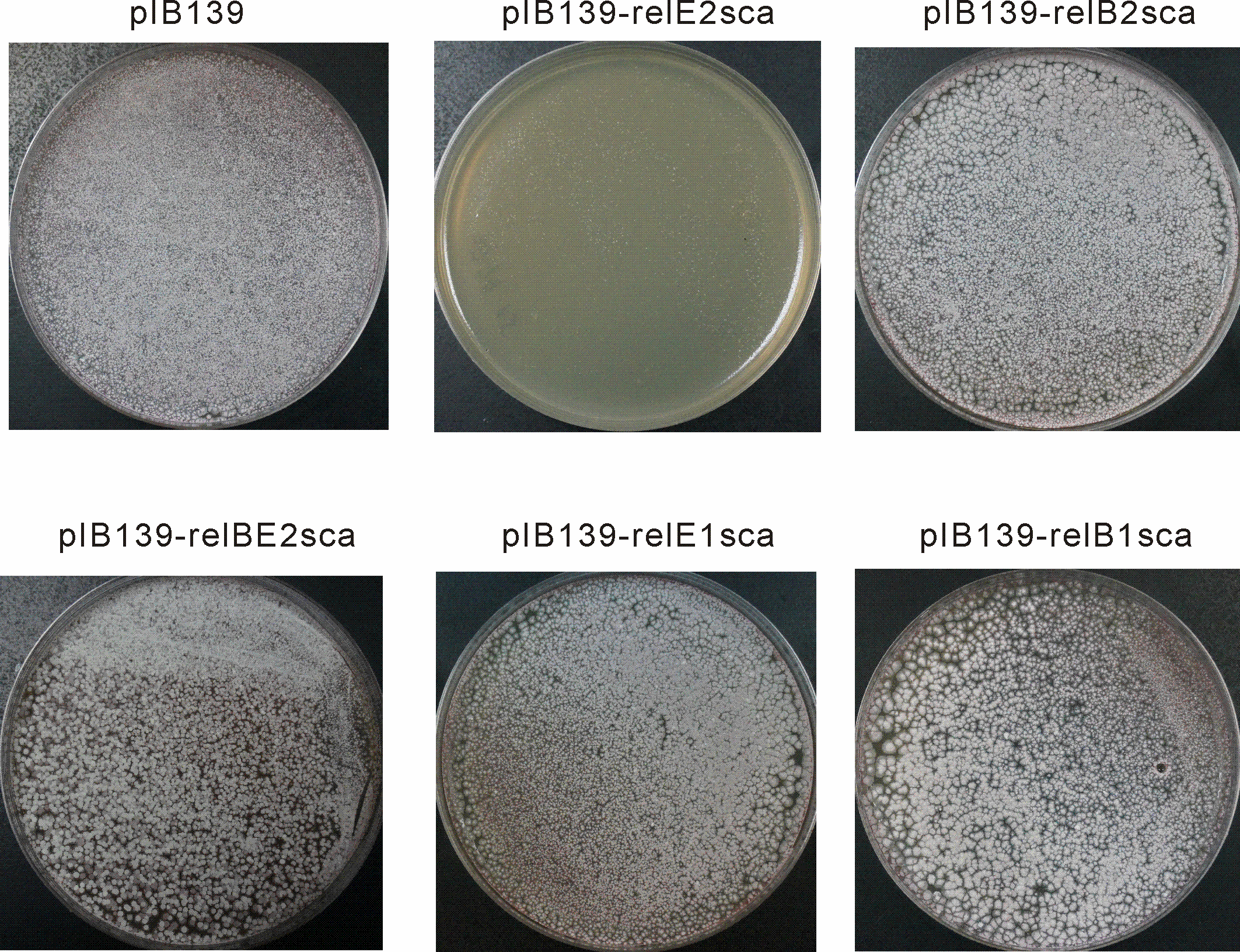


**Supplementary Figure S2.** Effect of the *S. cattleya* RelBE proteins (RelE1sca, RelB1sca, RelE2sca, and RelB2sca) on the growth of wild-type *S. lividans* TK24. MS plates showed the conjugants obtaining in conjugation with individual plasmids (supplementary Table S2), including the empty integrative plasmid pIB139, the plasmid carrying the *relE1sca* (pIB139-relE1sca), the plasmid carrying the *relB1sca* (pIB139-relB1sca), the plasmid carrying the *relB2sca* (pIB139-relB2sca), the plasmid carrying the *relE2sca* (pIB139-relE2sca) and the plasmid carrying *relBE2sca* module (pIB139-relBE2sca).


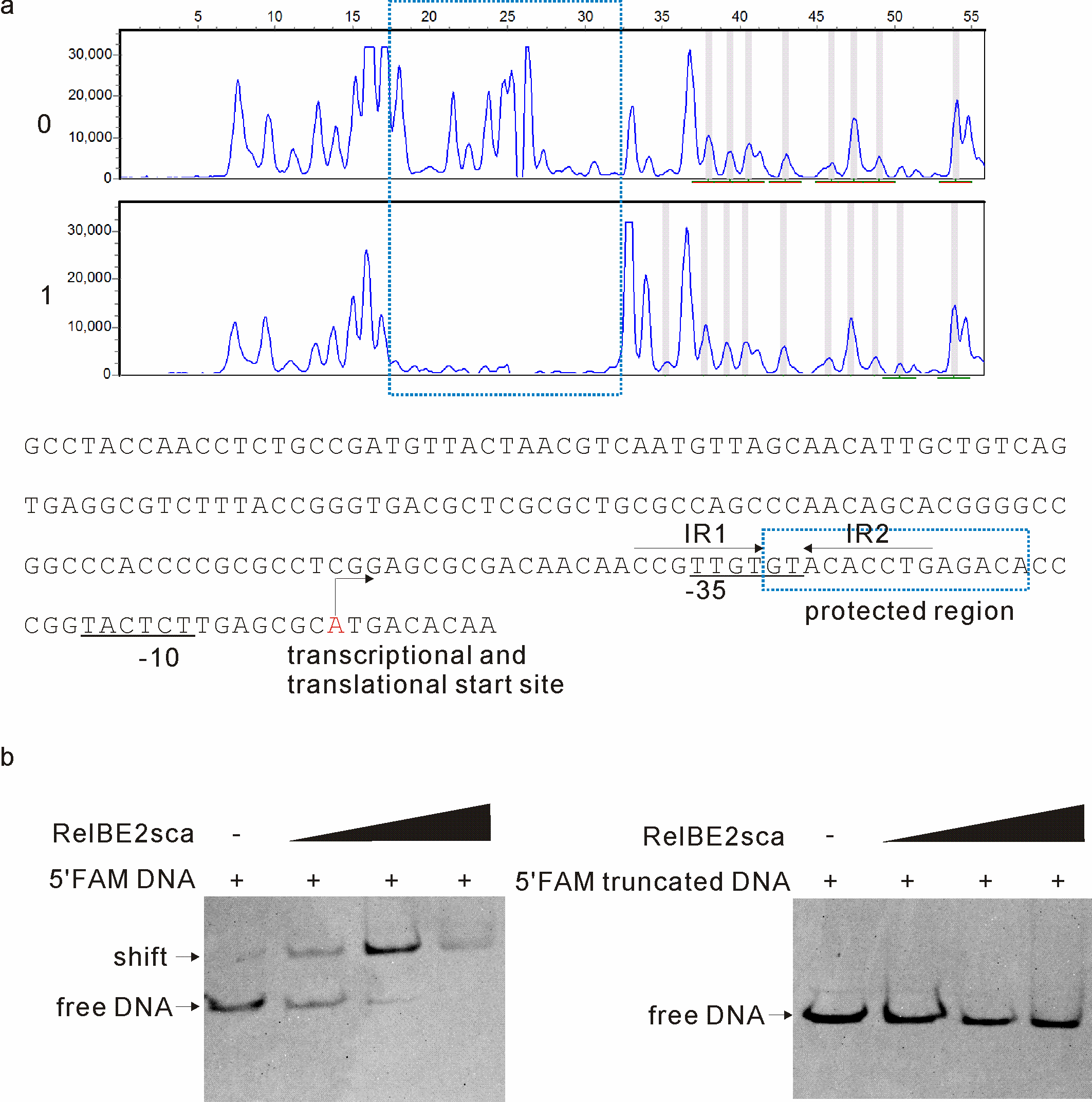


**Supplementary Figure S3.** DNase I footprinting assay for locating the binding sites of *S. cattleya* RelBE2sca complex into P*_relBE2_*, the promoter region of *relBE2sca*. (a) DNA sequencing Electropherogram based visualization for protection pattern of P*_relBE2_* after digestion with DNase I following the incubation with (in the bottom, 0) or without (on the top, 1) His6-RelBE2sca complex. The fluorescence signal of the FAM-labelled DNA fragments was plotted against the sequence length of the fragment. The protected DNA region containing inverted repeat (IR) was shown in the blue box. (b) The increased His6-RelBE2sca complex was incubated with 5’-FAM-labeled promoter. Left, wild type; right, mutant with the deletion of the protected DNA region.





**Supplementary Figure S4**. Transcription of the antitoxin gene *relB2sca* and the ClpP proteinase genes under different environmental stresses. These stress factors included: (a) osmotic pressure; (b) high temperature of 37℃; (c) strong amino acid starvation with addition of 0.4 mg/ml DL-serine hydroxamate; (d) glucose starvation with addition of 1% methyl-a-D-glucopyranoside; (e) antibiotic treatment with addition of 30 μg/ml chloramphenicol; (f) SOS response with induction of 1 μg/ml mitomycin C. Gene transcription level was measured by using real-time quantitative PCR. The experiments were repeated three times, and the error bars represented the standard deviation.


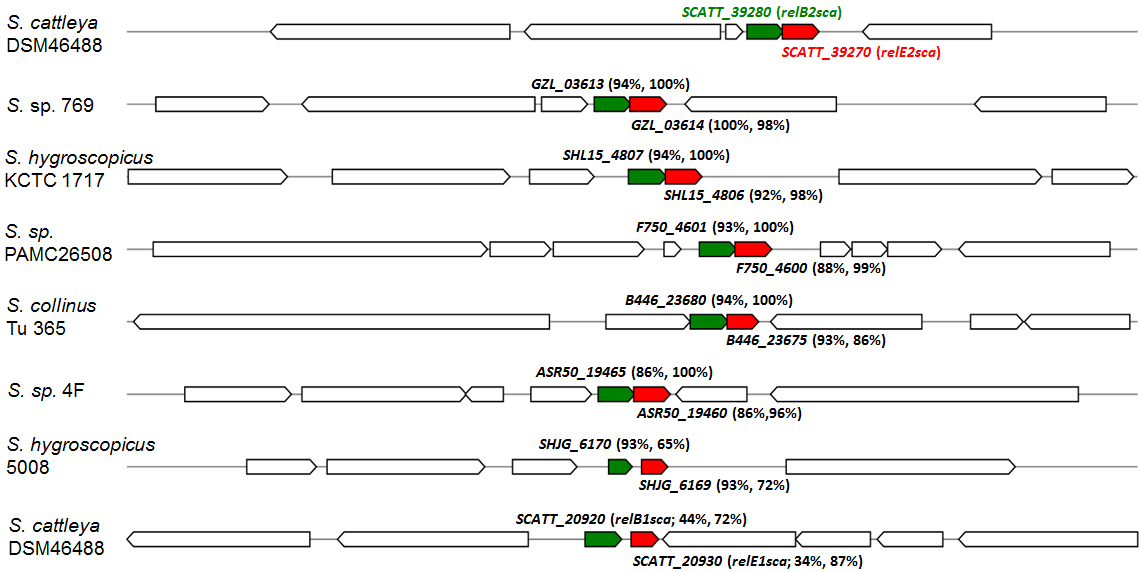


**Supplementary Figure S5.** Alignment maps of the *relBE* type II toxin-antitoxin loci found on the completely sequenced *Streptomyces* chromosomes. The BLASTp searches + hit collocation approach was used to generate the alignment. The matching genes are shown as color-matched: red, the toxin gene *relE*; green, the antitoxin gene *relB*. The locus tag names are followed by parentheses to indicate the BLASTp identities and coverage against the RelB2sca or RelE2sca protein of *S. cattleya* DSM46488.


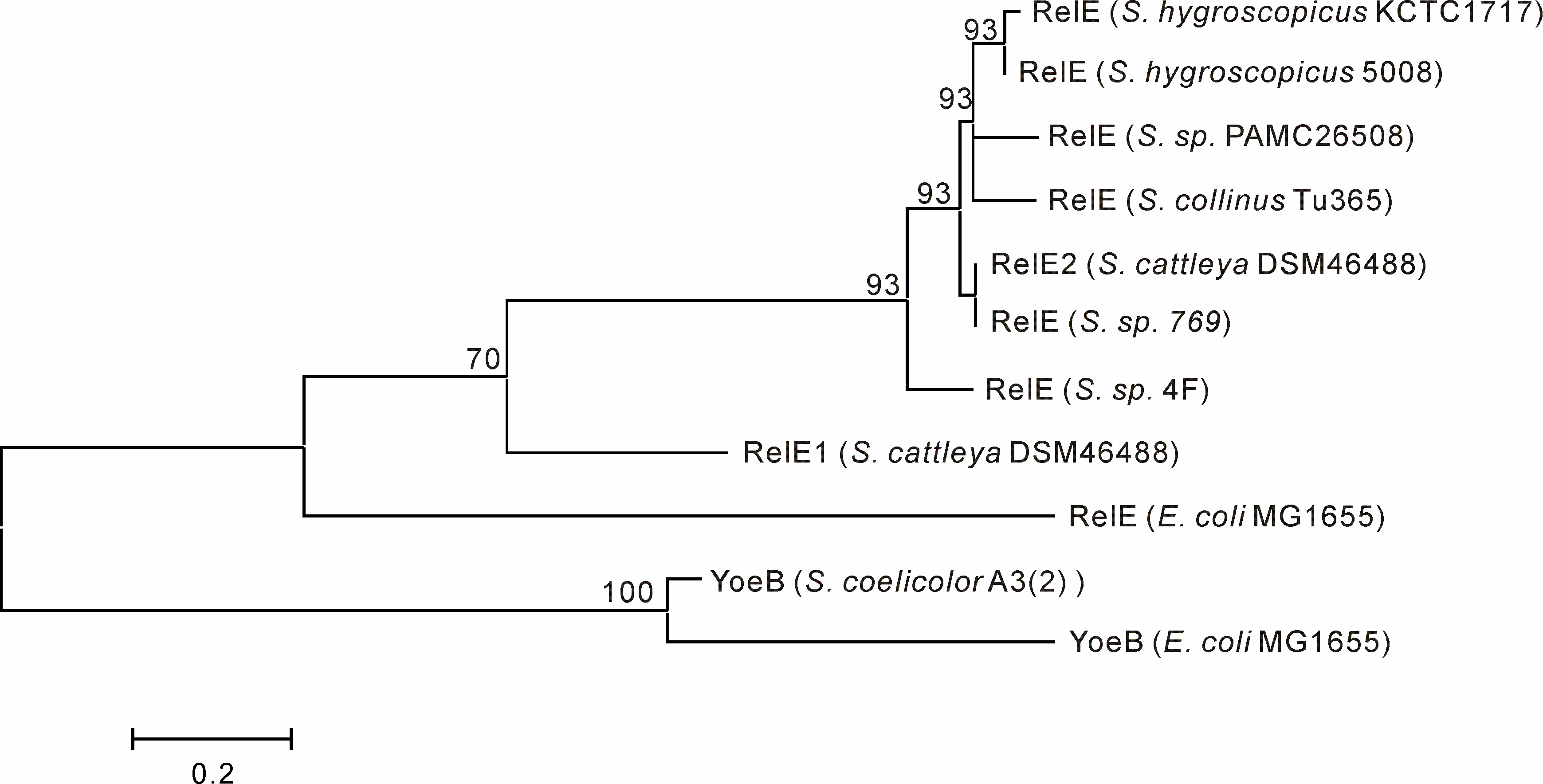


**Supplementary Figure S6.** Inferred phylogenetic relationships of the 9 RelE and 2 YoeB toxin proteins of *Streptomyces* and *E. coli*. The Maximum Likelihood tree was generated with MEGA7. Bootstrap percentage values (500 replicates) are shown at the nodes[^7^](#_ENREF_7). Species names are as indicated as following the RelE protein names.


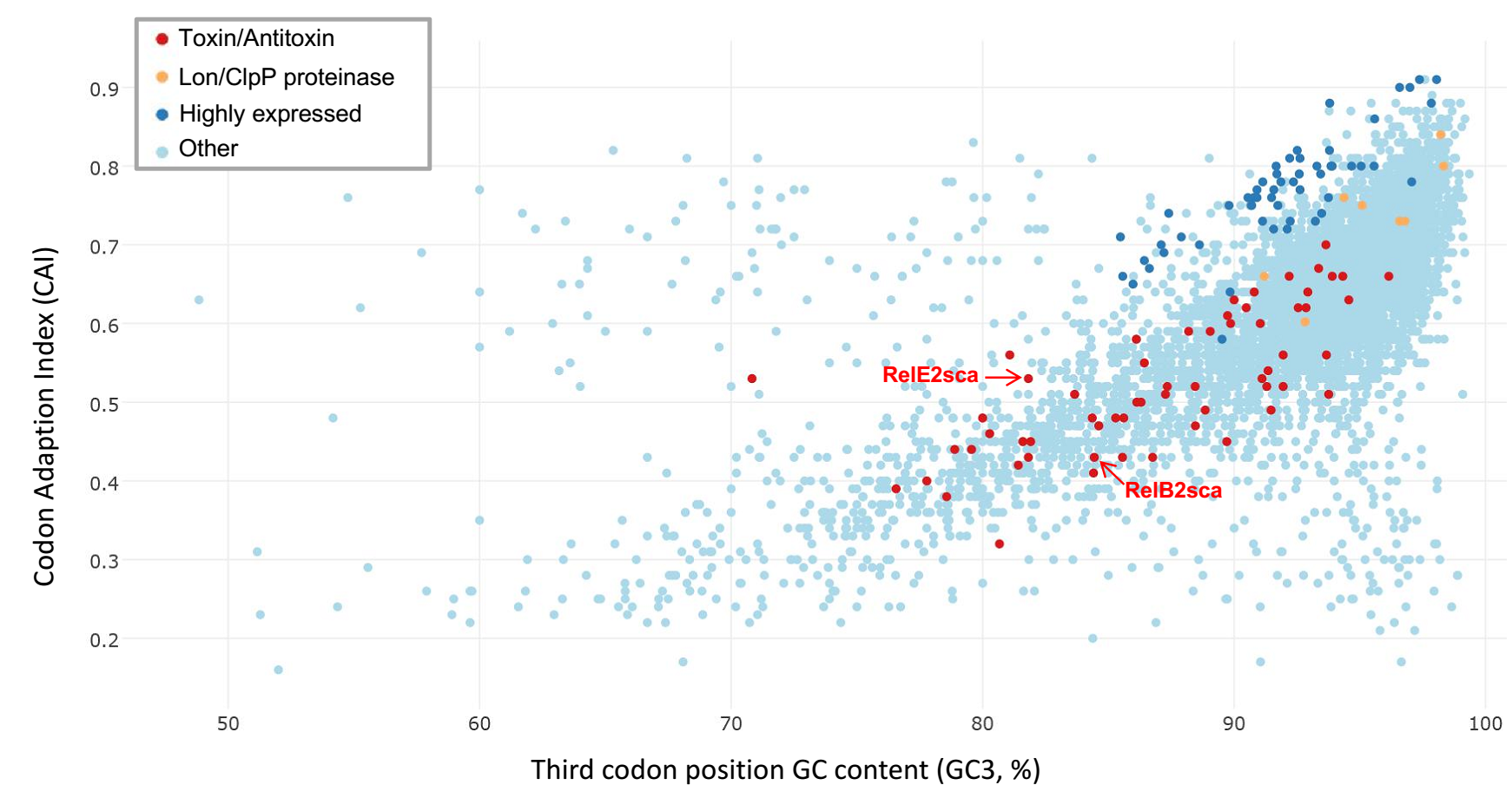


**Supplementary Figure S7.** Scatter plots of Codon Adaption Index (CAI) values against the third codon position GC content (GC3) values for the 5822 annotated genes on the *S. cattleya* DSM46488 chromosome[^8^](#_ENREF_8). The CAI values (y-axis) was computed on the reference set of 55 highly expressed genes (blue dots) (HEGS), coding for translation elongation factors Tu (SCATT_35610), Ts (SCATT_44440) and G (SCATT_35600) in addition to 52 ribosomal proteins [Sharp, 2005]. The red and yellow dots correspond to the genes coding for toxin/antitoxin proteins and Lon/ClpP proteinases, respectively.


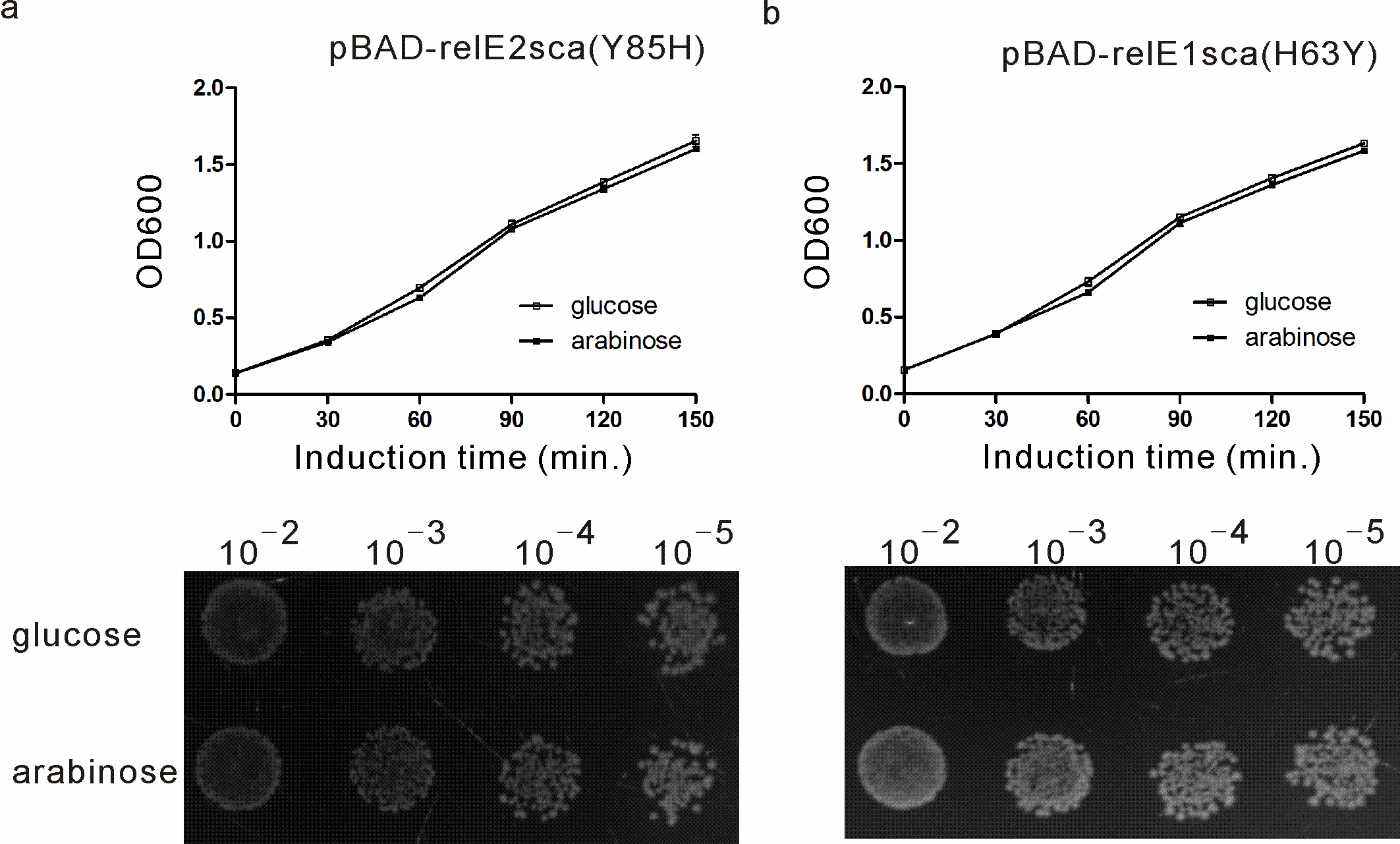


**Supplementary Figure S8**. Effect of over-expression of the RelE2sca Y85H mutant and RelE1sca H63Y on the growth of *E. coli* MGJ9587. Cells carrying plasmid pBAD-relE2sca(Y85H) (a) or pBAD-relE1sca(H63Y) (b) were grown in LB medium until OD600 reached 0.2, then the cultures were divided into two equal parts. At the time zero, 0.2% glucose (indicated by hollow square) was added into one part, and 0.2% L-arabinose (indicated by solid square) was added into the other half. Cell growth was monitored by measuring the OD600 every 30 minutes. The means and standard deviation of three different experiments were present. 3 µl of serial dilutions of different cultures which were collected after induced by glucose or arabinose for 1 hour were spread onto the LA plate and incubated at 37℃ for 12 hours.


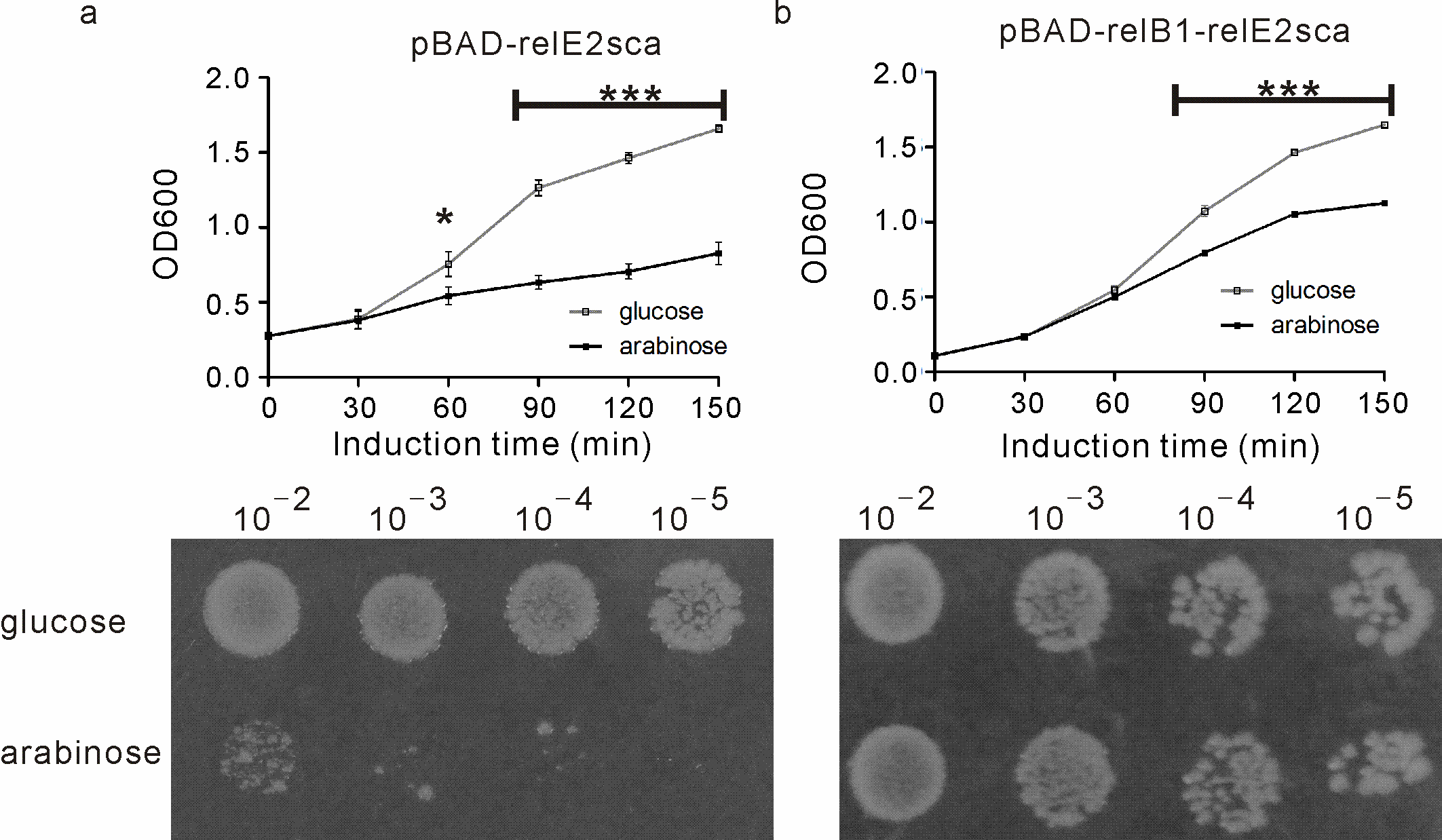


**Supplementary Figure S9.** Interaction of the relE2sca toxin and the relB1sca antitoxin in *S. cattleya*. *E. coli* MGJ5987 transformed with individual plasmids were grown in LB medium until the OD600 reached 0.2: (a) control plasmid pBAD-relE2sca, (b) plasmid pBAD-relB1sca-relE2sca. Then, at time zero, 0.2% glucose (the hollow square) was added to one-half of each culture and 0.2% L-arabinose (the solid square) was added to the other half. Cell growth was monitored by measuring the OD600 every 30 minutes. The means and standard deviation of three different samples were present. For statistical analysis, two-way analysis of variance with Bonferroni post-tests were used to obtain P values for each time point: *, P < 0.05; **, P < 0.01; ***, P < 0.001. 3 µl of serial dilutions of different cultures which were collected after induced by glucose or arabinose for 1 hour were spread onto the LA plate and incubated at 37℃ for 12 hours.


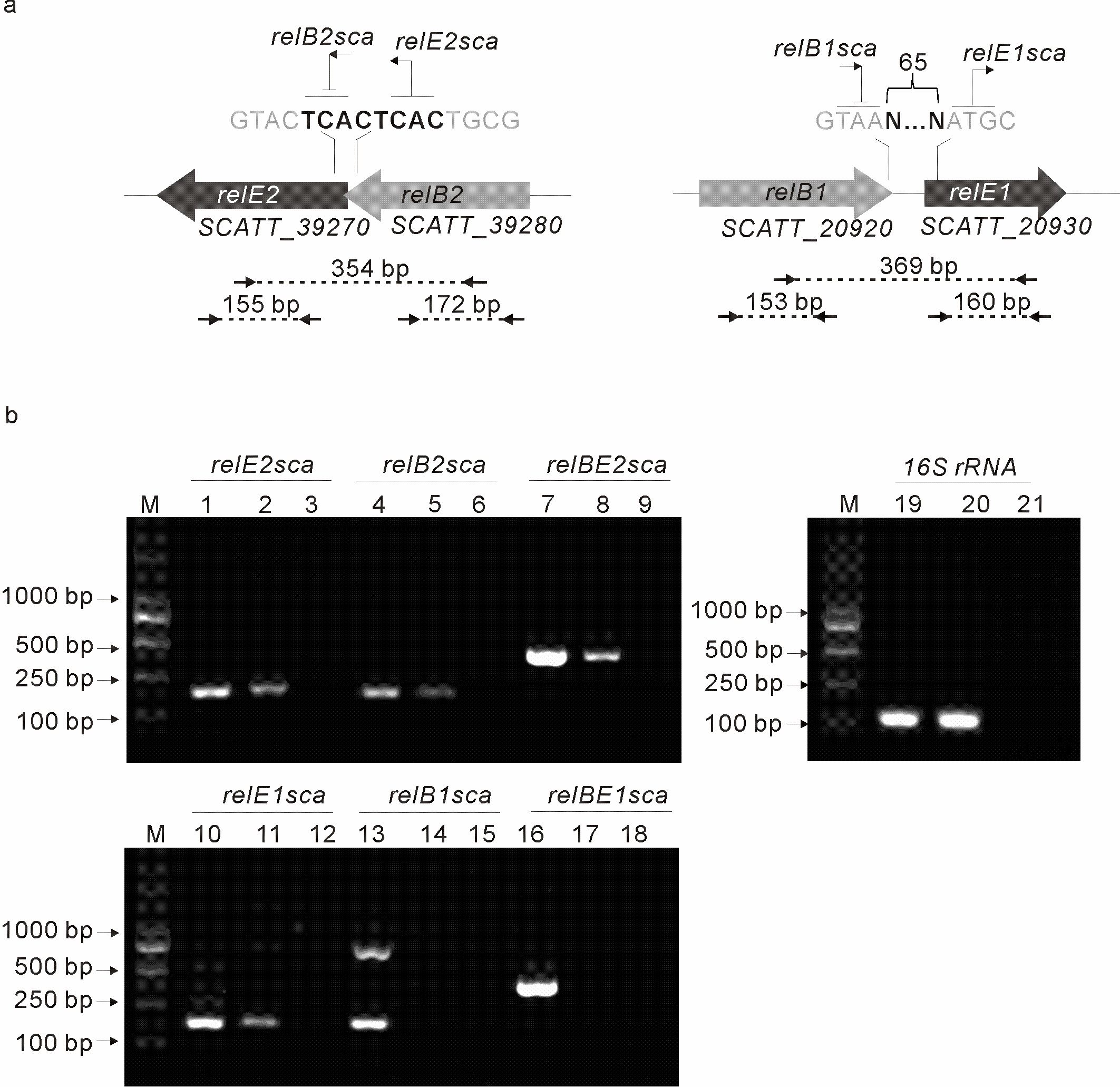


# **Supplementary Figure S10.** Original images of cropped gels used in Fig. 2. Genetic organization of the two putative *relBE* loci on *S. cattleya* DSM46488 chromosome: *relBE1sca* (*SCATT_20920-SCATT_20930*) and *relBE2sca* (*SCATT_39280-SCATT_39270*). (a) Scaled schematic representation of the toxin and antitoxin genes. The overlapping or separate region is shown in bold. The arrows indicate the primers used in the transcription analysis and the numbers indicate the expected size of the PCR products; (b) Transcription analysis of PCR amplification of *S. cattleya relBE* genes using cDNA and gene-specific primers to amplify from the 3’ end of *relBs* to the 5’ end of *relEs* (spanning both the protein-coding and the intergenic region). Lane M indicates the standard DNA size marker. Each set of the three lanes consisted of positive controls using genomic DNA as template (gDNA) , PCR amplified products using cDNA prepared from log-phase *S. cattleya* (cDNA) and negative controls using the total RNA without reverse transcriptase (RNA). 16S rRNA was used as the positive control.

**References**

1 Maisonneuve, E., Shakespeare, L.J., Jørgensen, M.G. & Gerdes, K. Bacterial persistence by RNA endonucleases. *Proceedings of the National Academy of Sciences of the United States of America* **108**, 13206-13211 (2011).

2 Paget, M.S.B., Chamberlin, L., Atrih, A., Foster, S.J. & Buttner, M.J. Evidence that the extracytoplasmic function sigma factor sigmaE is required for normal cell wall structure in *Streptomyces coelicolor* A3(2). *Journal of Bacteriology* **181**, 204-211 (1999).

3 Hopwood, D. A., Kieser, T., Wright, H.M. & Bibb, M.J. Plasmids, recombination and chromosome mapping in *Streptomyces lividans* 66. *Journal of General Microbiology* **129**, 2257-2269 (1983).

4 Zhao, C. *et al.* Insights into fluorometabolite biosynthesis in *Streptomyces cattleya* DSM46488 through genome sequence and knockout mutants. *Bioorganic Chemistry* **44**, 1-7 (2012).

5 Wilkinson, C. J. *et al.* Increasing the efficiency of heterologous promoters in actinomycetes. *Journal of Molecular Microbiology & Biotechnology* **4**, 417-426 (2002).

6 He, Y. *et al.* Two pHZ1358-derivative vectors for efficient gene knockout in *Streptomyces*. *Journal of Microbiology & Biotechnology* **20**, 678-682 (2010).

7 Kumar, S., Stecher, G. & Tamura, K. MEGA7: Molecular evolutionary genetics analysis version 7.0 for bigger datasets. *Molecular Biology and Evolution* (2016).

8 Sharp, P. M., Elizabeth, B., Grocock, R.J., Peden, J.F. & R Elizabeth, S. Variation in the strength of selected codon usage bias among bacteria. *Nucleic Acids Research* **33**, 1141-1153 (2005).
